# The Notch1/CD22 signaling axis disrupts Treg function in SARS-CoV-2–associated multisystem inflammatory syndrome in children

**DOI:** 10.1172/JCI163235

**Published:** 2023-01-03

**Authors:** Mehdi Benamar, Qian Chen, Janet Chou, Amélie M. Julé, Rafik Boudra, Paola Contini, Elena Crestani, Peggy S. Lai, Muyun Wang, Jason Fong, Shira Rockwitz, Pui Lee, Tsz Man Fion Chan, Ekin Zeynep Altun, Eda Kepenekli, Elif Karakoc-Aydiner, Ahmet Ozen, Perran Boran, Fatih Aygun, Pinar Onal, Ayse Ayzit Kilinc Sakalli, Haluk Cokugras, Metin Yusuf Gelmez, Fatma Betul Oktelik, Esin Aktas Cetin, Yuelin Zhong, Maria Lucia Taylor, Katherine Irby, Natasha B. Halasa, Elizabeth H. Mack, Overcoming COVID-19 Investigators, Sara Signa, Ignazia Prigione, Marco Gattorno, Nicola Cotugno, Donato Amodio, Raif S. Geha, Mary Beth Son, Jane Newburger, Pankaj B. Agrawal, Stefano Volpi, Paolo Palma, Ayca Kiykim, Adrienne G. Randolph, Gunnur Deniz, Safa Baris, Raffaele De Palma, Klaus Schmitz-Abe, Louis-Marie Charbonnier, Lauren A. Henderson, Talal A. Chatila

**Affiliations:** 1 Division of Immunology, Boston Children’s Hospital, Boston, Massachusetts, USA.; 2 Department of Pediatrics, Harvard Medical School, Boston, Massachusetts, USA.; 3 Department of Biostatistics, Harvard T.H. Chan School of Public Health, Boston, Massachusetts, USA.; 4 Brigham and Women’s Hospital, Department of Dermatology, Harvard Medical School, Boston, Massachusetts, USA.; 5 Unit of Clinical Immunology and Translational Medicine, IRCCS Ospedale Policlinico San Martino, Genoa, Italy.; 6 Department of Cardiology, Boston Children’s Hospital, Boston, Massachusetts, USA.; 7 Division of Pulmonary and Critical Care, Massachusetts General Hospital, Boston, Massachusetts, USA.; 8 Department of Medicine, Harvard Medical School, Boston, Massachusetts, USA.; 9 The Manton Center for Orphan Disease Research, Boston Children’s Hospital, Boston, USA.; 10 Ministry of Healthy, Marmara University Education and Training Hospital, Department of Pediatrics, Istanbul, Turkey.; 11 Marmara University, Faculty of Medicine, Division of Pediatric Infectious Diseases, Istanbul, Turkey.; 12 Division of Pediatric Allergy and Immunology, The Isil Berat Barlan Center for Translational Medicine, Istanbul, Turkey.; 13 Marmara University, Faculty of Medicine, Division of Social Pediatrics, Istanbul, Turkey.; 14 Division of Pediatric Allergy and Immunology, Faculty of Medicine, Istanbul University-Cerrahpasa, Istanbul, Turkey.; 15 Department of Immunology, Aziz Sancar Institute of Experimental Medicine (Aziz Sancar DETAE), Istanbul University, Istanbul, Turkey.; 16 Arkansas Children’s Hospital, Little Rock, Arkansas, USA.; 17 Division of Pediatric Infectious Diseases, Department of Pediatrics, Vanderbilt University Medical Center, Nashville, Tennessee, USA.; 18 Division of Pediatric Critical Care Medicine, Medical University of South Carolina, Charleston, South Carolina, USA; 19 A complete list of the Overcoming COVID-19 Investigators is provided in the Supplemental Acknowledgments.; 20 DINOGMI, Università degli Studi di Genova, Genova, Italy and Center for Autoinflammatory Diseases and Immunodeficiencies, IRCCS Istituto Giannina Gaslini, Genova, Italy.; 21 Center for Autoinflammatory Diseases and Immunodeficiencies, IRCCS Istituto Giannina Gaslini, Genova, Italy.; 22 Clinical and Research Unit of Clinical Immunology and Vaccinology, Bambino Gesù Children’s Hospital, IRCCS, Rome, Italy.; 23 Chair of Pediatrics, Department of Systems Medicine, University of Rome “Tor Vergata,” Roma, Italy.; 24 Department of Cardiology, Boston Children’s Hospital, Boston, Massachusetts, USA.; 25 Division of Newborn Medicine and Genetics and Genomics, Department of Pediatrics, Boston Children’s Hospital, Harvard Medical School, Boston, Massachusetts, USA.; 26 Division of Pediatric Allergy and Immunology, Faculty of Medicine, Istanbul University-Cerrahpasa, Istanbul, Turkey.; 27 Department of Anesthesiology, Critical Care, and Pain Medicine, Boston Children’s Hospital, Boston, Massachusetts, USA.; 28 Department of Internal Medicine (DIMI), University of Genoa, Genoa, Italy.; 29 CNR Institute of Biomolecular Chemistry (IBC), Pozzuoli, Napoli, Italy.

**Keywords:** COVID-19, Immunology, Adaptive immunity, T cells, Tolerance

## Abstract

Multisystem inflammatory syndrome in children (MIS-C) evolves in some pediatric patients following acute infection with SARS-CoV-2 by hitherto unknown mechanisms. Whereas acute-COVID-19 severity and outcomes were previously correlated with Notch4 expression on Tregs, here, we show that Tregs in MIS-C were destabilized through a Notch1-dependent mechanism. Genetic analysis revealed that patients with MIS-C had enrichment of rare deleterious variants affecting inflammation and autoimmunity pathways, including dominant-negative mutations in the Notch1 regulators *NUMB* and *NUMBL* leading to Notch1 upregulation. Notch1 signaling in Tregs induced CD22, leading to their destabilization in a mTORC1-dependent manner and to the promotion of systemic inflammation. These results identify a Notch1/CD22 signaling axis that disrupts Treg function in MIS-C and point to distinct immune checkpoints controlled by individual Treg Notch receptors that shape the inflammatory outcome in SARS-CoV-2 infection.

## Introduction

COVID-19, caused by the severe acute respiratory syndrome coronavirus 2 (SARS-CoV-2), has resulted in massive morbidity and mortality worldwide (1, 2). Acute infection is associated in some individuals with pneumonia and marked hypoxia, leading to acute respiratory distress syndrome as well as other life-threatening complications (3–5). This inflammation critically involves a dysregulated immune response characterized by intense activation of innate and adaptive immunity associated with features of a cytokine storm (6, 7). While most patients recover from this acute infection, a subset develops persistent symptoms related to dysfunction in various organ systems including the respiratory, cardiovascular, gastrointestinal (GI), renal, and central nervous systems (8).

A special case in point is the course of SARS-CoV-2 infection in children. Although most children remain asymptomatic or develop mild infection, some develop a multisystem inflammatory syndrome in children (MIS-C) approximately 1 month after initial infection (9–13). These patients exhibit severe immune dysregulation characterized by intense cytokine production and lymphocyte activation associated with fever and end-organ dysfunction including mucocutaneous, cardiovascular, hematologic, and, especially, GI systems (14–21). IFN-γ has been identified as a key cytokine in MIS-C, with increased levels being associated with disease severity and organ system involvement (22–25). The patients also exhibit robust T cell activation with T cell receptor (TCR) repertoire skewing (24–29). There are defining characteristics of MIS-C that remain perplexing, including the substantial delay between the initial SARS-CoV-2 infection and the onset of MIS-C (9, 10, 12). Unlike children with acute COVID-19 pneumonia, most patients with MIS-C were previously healthy and were able to mount a robust immune response to SARS-CoV-2 with neutralizing antibodies against the virus (11, 18, 30). This constellation of features in MIS-C suggests that an evolving hyperinflammatory immune response to SARS-CoV-2 is part of the pathophysiology of this syndrome. Indeed, studies based on relatively small numbers of patients suggest that a genetic predisposition may contribute to the immune dysregulation in MIS-C (31, 32).

Notch signaling pathways have emerged as important regulators of the immune system by influencing both Tregs and conventional T (Tconv) cell responses (33, 34). In mammals, the Notch family is composed of 4 Notch receptors (Notch1–4) and 5 ligands (Delta-like 1, -3, and -4 and Jagged 1 and -2) (35). Recent studies have outlined a prominent role for Notch4 in the immune dysregulation in acute COVID-19 and related respiratory viral illnesses (36). Notch4 is upregulated on lung tissue Tregs in an IL-6–dependent manner to subvert their tissue repair function in favor of an inflammatory response (36–39). The *NOTCH4* locus is associated with critical illness in patients with COVID-19 (40). However, the immune-dysregulatory mechanisms operative in post-acute COVID-19 syndromes, including MIS-C, remain unclear.

In this study, we show that patients with MIS-C had robust T cell activation in association with increased Notch signaling in Tregs. In particular, while Notch4 was also upregulated on circulating Tregs in children with acute COVID-19 as a function of disease severity, the Tregs in those with MIS-C additionally showed upregulation of Notch1 expression, a pathway previously implicated in Th1-skewed immune dysregulation, autoimmunity, graft-versus-host disease, and solid organ rejection (41, 42). Gene enrichment using whole-genome/-exome sequence analysis with Fisher’s exact test and Monte Carlo simulation revealed in patients with MIS-C an enrichment of rare mutations affecting pathways of inflammation and autoimmunity, many of which contained Notch-related genes. Consistent with these results, loss-of-function mutations were identified in the negative Notch1 regulators *NUMB* and *NUMBL* (43). An in vitro experiment revealed that this loss-of-function mutation promoted Notch1 expression. Moreover, in mice either expressing an active form of Notch1 in Tregs (*Foxp3^EGFPCre^* *R26^N1c/+^*) or lacking NUMB expression in these cells (*Foxp3^EGFPCre^* *Numb^Δ/Δ^*), treatment with polyinosinic:polycytidylic acid (poly I:C) to simulate viral infection induced systemic inflammation. Notch1 signaling in Tregs induced the B cell–inhibitory receptor CD22 (44, 45), which promoted systemic inflammation in association with the expression of the α4β7 gut-homing receptor. CD22 destabilized Tregs and impaired their suppressive function in an mTORC1-dependent manner. Treatment of *Foxp3^EGFPCre^* *R26^N1c/+^* or *Foxp3^EGFPCre^* *Numb^Δ/Δ^* mice with an anti-CD22 mAb suppressed the development of systemic inflammation following poly I:C treatment by restoring the Treg-suppressive function. These findings point to the mobilization of Treg-specific tissue-inflammatory licensing modules involving different Notch receptors that is operative in MIS-C and suggest the possible benefit of interventions along the Notch1/CD22 axis as a therapeutic strategy in MIS-C.

## Results

### Increased CD4^+^ T cell activation and Treg destabilization in MIS-C.

To elucidate the immune-dysregulatory mechanisms operative in MIS-C, we studied an international cohort of 45 children with MIS-C and 50 children with COVID-19 from centers in the United States, Italy, and Turkey (Supplemental Table 1; supplemental material available online with this article; https://doi.org/10.1172/JCI163235DS1 and the *Patient cohorts* section in Methods). For comparison, 5 children with Kawasaki disease (KD), 12 adults with COVID-19, and 18 pediatric healthy controls were also evaluated. All patients with MIS-C met the CDC Case Definition for MIS-C (46), whereas 93% fulfilled the WHO case definition (47–49). Fever was universal in patients with MIS-C, and rash (49%), conjunctivitis (58%), and GI symptoms (96%) were also common. Children with MIS-C had high inflammation (median C-reactive protein [CRP] 16.0 mg/dL, IQR 7.8–24.0); lymphopenia (median absolute lymphocyte count of 0.91 × 10^3^/mL, IQR 0.53–1.35); and coagulopathy (median D-dimer 3.1 mcg/mL, IQR 1.5–6.2). Over 90% of patients with MIS-C had positive SARS-CoV-2 serologies. A total of 18 of 45 (40%) were considered to have severe MIS-C, defined by admission to the intensive care unit (ICU), the need for vasopressor support, and/or the development of coronary artery aneurysms. The demographics and key clinical findings in the respective patient groups are delineated in Supplemental Table 1.

**Figure 1:**
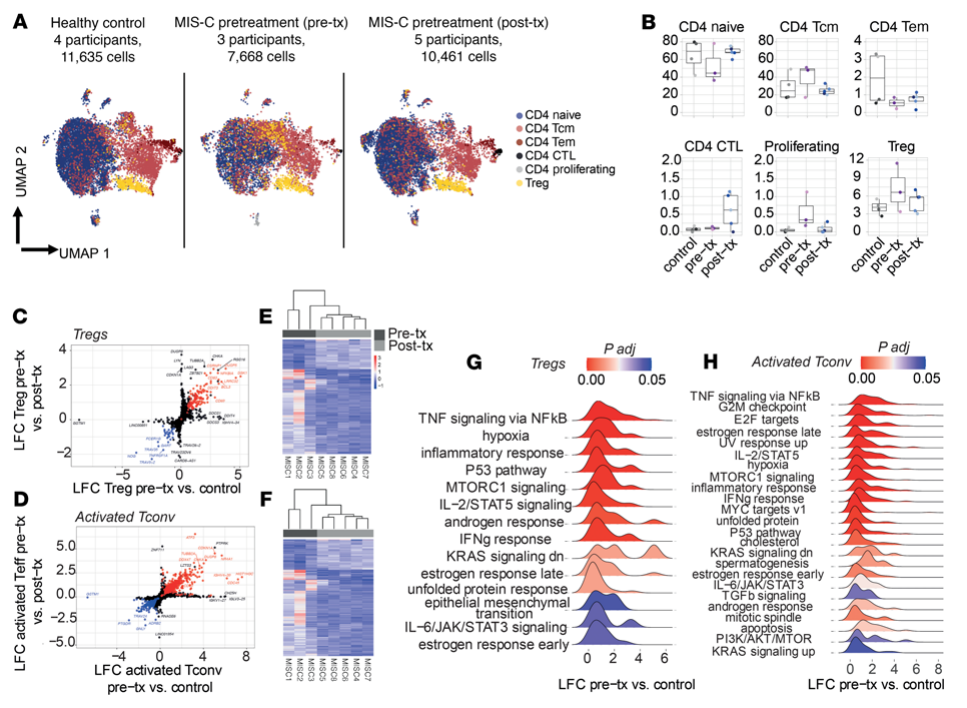
Increased CD4^+^ T cell activation and Treg destabilization in MIS-C. Circulating CD4^+^ T cells from control and pre- and post-treatment MIS-C individuals were studied with 10x Genomics. (**A**) Uniform manifold approximation and projection (UMAP) of normalized and harmonized data set, split by disease group and color coded by cell type. Cell identities were defined by mapping the data to a reference human PBMC data set with Azimuth. (**B**) Frequencies (percentage) of each cell type among total CD4^+^ T cells for each patient, as determined with scRNA-Seq. (**C** and **D**) Data on log_2_ fold change (LFC) in gene expression derived from independent pseudobulk DEA of pretreatment MIS-C patients versus healthy controls (*x* axis) and of pretreatment MIS-C versus post-treatment MIS-C patients (*y* axis) in Tregs (**C**) and activated Tconv cells (**D**). For each cell type, genes differentially expressed (*P* < 0.2) in pretreatment MIS-C versus both control and post-treatment individuals are highlighted (blue: LFC <0, red: LFC >0). (**E** and **F**) Heatmaps of all genes found to be significantly (*P* < 0.05) differentially expressed in Tregs (**E**) and Tconv cells (**F**) according to pseudobulk DEA comparing results for pretreatment MIS-C versus control and pretreatment MIC-C versus post-treatment individuals. (**G** and **H**) LFC distributions of genes belonging to each of the corresponding enriched hallmarks. GSEA was run against the MSigDB hallmark database using ranked LFCs derived from pseudobulk DEAs of Tregs from pretreatment MIS-C patients versus controls. adj, adjusted; tx, treatment.

To further delineate the CD4^+^ T cell dynamics in MIS-C, we carried out single-cell RNA-Seq (scRNA-Seq) analysis on CD4^+^ T cells from the peripheral blood of 4 healthy controls, 3 patients with MIS-C sampled prior to treatment, and another 5 patients with MIS-C sampled after treatment. We first mapped our transcriptomic data to a reference human PBMC data set using Azimuth (50), thereby delineating 6 subsets of CD4^+^ T cells (Figure 1, A and B). We further performed a graph-based clustering analysis using Seurat, which uncovered 16 clusters. Eight of these clusters (clusters 1–8) were enriched in cells annotated as CD4 naive by Azimuth and expressing genes associated with a naive CD4^+^ T cell profile (e.g., *CCR7* and *SELL*), and 5 (clusters 10–14) were enriched in activated CD4^+^ T cells (*CD69*), including 1 with high NF-κB signaling (cluster 10; *NFKB1*). The final 3 clusters encompassed a mix of naive and activated cells, including 1 cluster delineated by virus-sensing gene transcripts (cluster 9; *IFIT2*, *IFIT3*), 1 cluster enriched in Treg transcripts (cluster 15; *FOXP3*), and another with mitotic cells (cluster 16; *TRBC1*) (Supplemental Figure 1, A–F). Prior to treatment, patients with MIS-C exhibited prominent expansion of cluster 10, enclosing both cells annotated as Tconv cells and Tregs by Azimuth. Cluster 10 was characterized by increased *NFKB1* expression and NF-κB signaling and contracted following immunomodulatory therapy (Supplemental Figure 1, A–F).

To further decipher differences in CD4^+^ T cell transcriptomic programs between patient groups, we also performed pseudobulk differential expression analysis (DEA) with a focus on both Tregs (cells found in cluster 15 or delineated as Tregs by Azimuth) and Tconv cells (cells found in clusters 9–14 and delineated as activated Tconv cells by Azimuth). We aggregated gene expression data at the patient level for Tregs and activated Tconv cells and performed pairwise comparisons of MIS-C pretreatment, post-treatment, and control groups using DESeq2. The DEAs were followed by gene set enrichment analyses (GSEA) against the MSigDB Hallmark collection and using the ranked log_2_ fold changes as input, which reinforced our prior observations of NF-κB pathway activation in pretreatment MIS-C samples, not only in Tconv cells but also in Tregs (Figure 1, C–H, and Supplemental Figure 1, G and H). Pathways that were up regulated in the MIS-C pretreatment group included mTORC1, whose hyperactivity has been previously noted to mediate Treg destabilization (Figure 1, C–H, and Supplemental Figure 1, G and H) (51, 52). These results indicated that MIS-C is associated with enhanced Tconv activation and Treg dysregulation.

### Increased NOTCH1 expression on CD4^+^ Tregs and Tconv cells in MIS-C.

**Figure 2:**
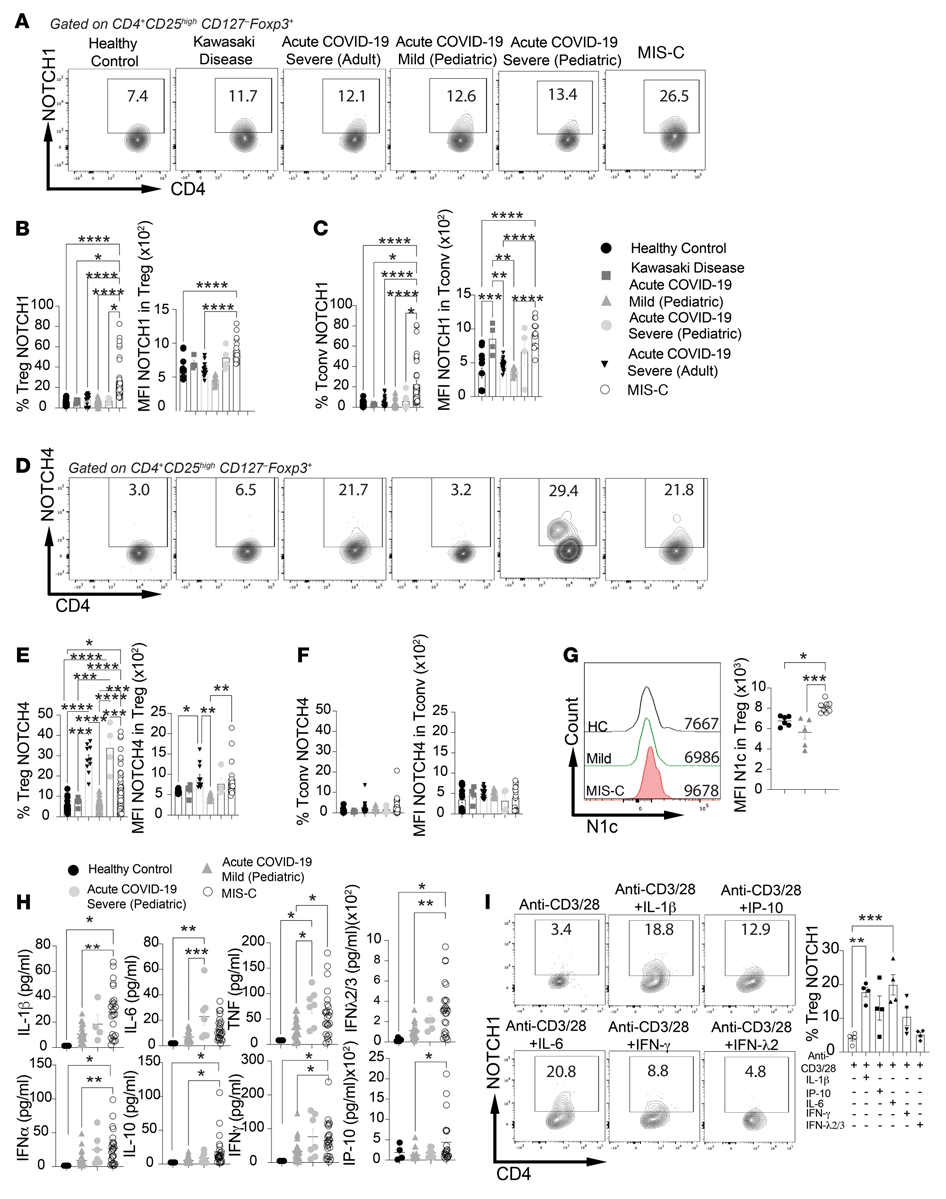
Increased Notch1 expression on circulating CD4^+^ Tregs and Tconv cells in MIS-C. (**A**–**F**) Flow cytometric analysis, cell frequencies, and MFI of Notch1 (**A**–**C**) and Notch4 (**D**–**F**) expression in CD4^+^ Tregs and Tconv cells from healthy controls, patients with KD, adult patients with severe COVID-19, pediatric patients with mild or severe COVID-19, and patients with MIS-C. (**G**) Flow cytometric analysis and MFI of N1c in CD4^+^ Tregs from healthy controls (HC) and pediatric patients with severe COVID-19 or MIS-C. (**H**) serum concentrations of IL-1β, IL-6, TNF, IFN-α, IFN-λ2/3, IFN-γ IL-10, and IP-10 in control and the respective patient group individuals. (**I**) Flow cytometric analysis and frequencies of Notch1 expression on anti-CD3– and anti-CD28–activated CD4^+^ human Tregs treated with the indicated cytokines. Each symbol represents 1 individual. Numbers in flow plots indicate percentages. Error bars indicate the SEM. *P < 0.05, **P < 0.01, ***P < 0.001, and ****P < 0.0001, by 1-way ANOVA with Dunnett’s post hoc analysis (**A**–**E**, **H**, and **I**).

Previous studies have demonstrated a key role for Notch signaling–mediated Treg dysregulation in licensing tissue inflammation (36, 38, 41, 42). For example, Notch4 is upregulated in lung tissue Tregs during SARS-CoV2 and influenza infections, leading to enhanced tissue inflammation and disease severity (36). We analyzed the expression of different Notch receptors on CD4^+^ Tregs and Tconv cells in pediatric patients with mild or severe COVID-19 and in those with MIS-C. As comparison groups, we included healthy children, adults with severe COVID-19, and children with KD, some of whose clinical features overlapped with those of the MIS-C patients (16, 31, 48, 49). There was a marked increase in Notch1 expression on both Tregs and Tconv cells from patients with MIS-C but not on those from the other groups (Figure 2, A–C, and Supplemental Figure 2A). Notch4 expression was also selectively increased on the circulating Tregs of adult and pediatric patients with severe COVID-19 or MIS-C but not on their Tconv cells. There was also no upregulation of Notch4 on Tregs of patients with mild COVID-19 or KD (Figure 2, D–F, and Supplemental Figure 2B). Notch1 expression in MIS-C was associated with increased intracellular expression of Notch1 cytoplasmic domain (N1c) in Tregs (Figure 2G). In contrast, Notch2 expression was increased on Notch1^+^ Tregs and Tconv cells of patients with MIS-C, albeit at a lower magnitude than that of Notch1, whereas there was no difference in the Notch2 single-positive Treg and Tconv cell populations between patients with MIS-C and healthy controls (Supplemental Figure 2C and Supplemental Figure 3, A, B, E, and F). Expression of Notch1 and Notch4 on Tregs of patients with MIS-C was nonoverlapping, suggesting that they may represent distinct Treg populations possibly arising in different tissues (Supplemental Figure 3, E and F). Also, there was no difference in Notch3 expression between the circulating Tregs and Tconv cells from the different patient populations and controls (Supplemental Figure 2D and Supplemental Figure 3, C and D). Overall, these results identified increased Notch1 expression on Tregs and Tconv cells as a distinguishing feature of pediatric patients with MIS-C.

Further analysis revealed that patients with MIS-C had a decrease in naive Tconv cells and Tregs associated with an increase in activated T cells (Supplemental Figure 4, A and B). As in previous studies (7), we found increased serum levels of IP-10, IL-1β, IL-6, and IFN-λ2/-3 in patients with MIS-C and in patients with severe COVID-19 compared with controls (Figure 2H). Also, Tregs and Tconv cells of patients with MIS-C versus those with severe COVID-19 or KD and control individuals had increased IFN-γ production (Supplemental Figure 4, C and D). Notably, IFN-γ expression was selectively increased in Tregs of patients with MIS-C, while IFN-γ expression in Tconv cells was common to both patients with severe COVID-19 and those with MIS-C (Supplemental Figure 4, C and D). We analyzed the capacity of different cytokines that were found to be increased in the sera of patients with MIS-C to induce Notch1 expression on cell-sorted CD4^+^CD25^+^CD127^–^ Tregs from control participants. IL-1β and IL-6, and to a lesser extent IFN-γ and IFN-γ–induced protein 10 (IP-10), all induced increased Notch1 expression on human Tregs (Figure 2I). MIS-C patients from North America and Europe were overall closely matched in their immunological analyses, with differences found only in Notch4, IP-10, and IFN-λ1 between these 2 cohorts (Supplemental Figure 5, A–F). Together, these results linked the upregulation of Notch1 expression on CD4^+^ Tregs and Tconv cells with the development of MIS-C.

### Identification of Notch pathway genetic variants in MIS-C.

**Figure 3:**
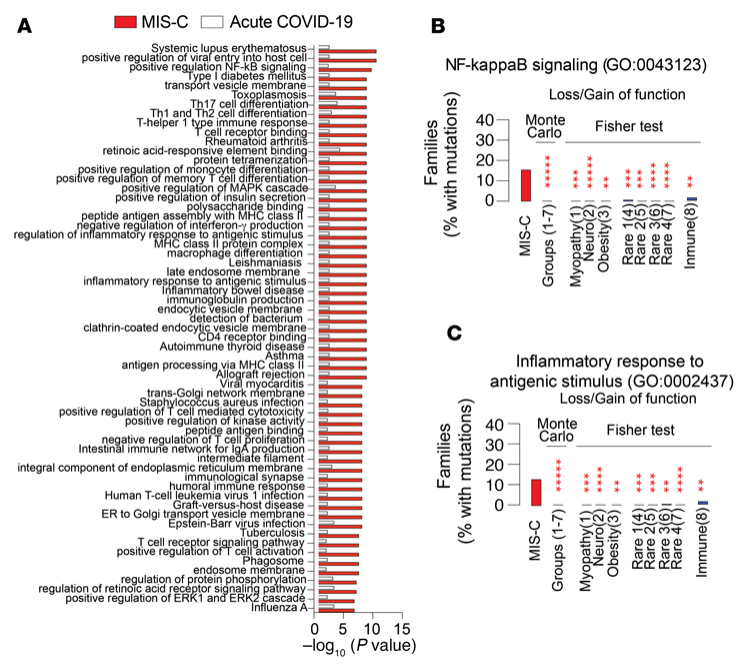
Identification of the genetic pathway operative in MIS-C. (**A**) KEGG and GO pathways differentially enriched in rare mutations in patients with MIS-C versus pediatric patients with acute COVID-19 (severe and mild) by Monte Carlo simulation and Fisher’s exact test as described in Methods. (**B** and **C**) Frequency of mutations in 2 representative pathways: “positive regulation of NF-κB signaling”(**B**) and “inflammatory response to antigenic stimulus” (**C**), identified in **A** versus other disease groups, either collectively by Monte Carlo simulation or individually by Fisher’s exact test. **P < 0.01, ***P < 0.001, ****P < 0.0001, and *****P < 0.00001, by Monte Carlo simulation and Fisher’s exact test.

To investigate underlying genetic factors that may predispose children to MIS-C versus acute pediatric COVID-19, we performed gene enrichment tests for rare variants (stop-gain/start-loss, frameshift deletions/insertions, and canonical splicing mutations) using 8,626 pathways from the Gene Ontology (GO) and Kyoto Encyclopedia of Genes and Genomes (KEGG) databases (8,299 and 327 pathways, respectively). We collected genome and exome sequences for 39 patients with MIS-C and 24 pediatric patients with acute COVID-19, which we compared with 8 different data sets comprising 4,682 exomes collected at the Boston Children’s Hospital, including exomes for 4 rare disease categories, obesity, myopathy, autism–attention deficit hyperaactivity disorder (autism-ADHD), and immune deficiency/dysregulation (53) (see Methods). All samples were processed using the Variant Explorer Pipeline (VExP) with the same set parameters to avoid bias in the selection of the rare variants (54). We performed a Fisher’s exact test for each group to assess enrichment in the respective GO and KEGG pathways. Furthermore, we validated these results by Monte Carlo simulation testing as an unbiased stochastic approach to test for enrichment in genetic variants along individual pathways in the MIS-C group versus the sum total of the clinical comparison groups used for the Fisher’s exact tests, as described in Methods. We found that several inflammation and autoimmunity pathways were significantly enriched in rare mutations (≤10 in 280,000 chromosomes) in patients with MIS-C compared to pediatric patients with COVID-19 patients or other comparison groups (Figure 3, A–C, and Supplemental Data File 1). A number of those pathways contained Notch-related genes. Specific Notch pathway mutations predicted to be damaging and linked to those pathways were identified and included *NOTCH2*, *NOTCH4*, and *RBPJL* (Supplemental Data File 2). Additionally, and in agreement with a previous report (55), we also detected rare heterozygous mutations in some genes, including *AP3B1, PRF1, LYST* and *DOCK8*, that are associated with familial hemophagocytic lymphohistiocytosis in patients with MIS-C (Supplemental Data File 2). Overall, these results indicated the presence of an underlying genetic predisposition to MIS-C.

**Figure 4:**
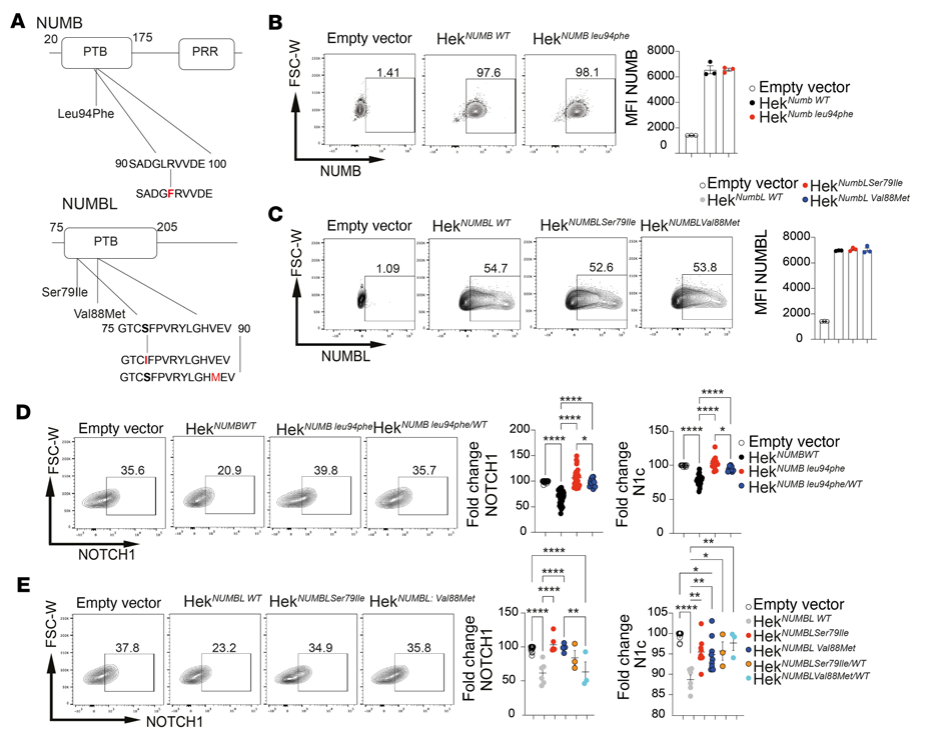
Identification of NUMB/NUMBL genetic variants in MIS-C. (**A**) Schematic representation of *NUMB* and *NUMBL* mutations identified in patients with MIS-C. (**B** and **C**) Expression of recombinant WT NUMB (NUMB^WT^) and NUMB^Leu94Phe^ (**B**), and NUMBL^WT^, NUMBL^Ser79Ile^, and NUMBL^Val88Met^ (**C**) proteins in NUMB/NUMBL-deficient HEK293 cells. (**D** and **E**) Flow cytometric analysis and fold change in expression of Notch1 and N1c in NUMB/NUMBL-deficient HEK293 cell transfected with NUMB^Leu94Phe^ protein (**D**) or NUMBL^Ser79Ile^ or NUMBL^Val88Met^ protein (**E**), either alone or together with the respective WT protein. Error bars indicate the SEM. *P < 0.05, **P < 0.01, and ****P < 0.0001, by 1-way ANOVA with Dunnett’s post hoc analysis (**B**–**E**). FSC-W, forward scatter width.

To validate the above findings from our initial cohort, we screened 88 additional patients with MIS-C from the US multicenter Overcoming COVID-19 Network for mutations in Notch-related genes (see Supplemental Methods) (9, 56). We identified rare damaging mutations in *NUMB* and *NUMBL*, which encode closely conserved eponymous proteins that negatively regulate Notch receptor signaling and trafficking and which are expressed by human Tregs and Tconv cells (Supplemental Figure 1, D and E) (43, 57–59). We further analyzed 3 mutations, found in different patients, that localized to the phosphotyrosine-binding (PTB) domain of *NUMB* (NM_001005745.1:c.280C>T; p.Leu94Phe) and *NUMBL* (NM_004756.5:c.236G>T, p.Ser79Ile; c.2 62G>A, p.Val88Met) (Figure 4A and Supplemental Data File 3). These mutations, which were either not found in the Genome Aggregation Database (gnomAD) (NUMB^Leu94Phe^ and NUMBL^Ser79IIe^; gnomAD = 0) or very rarely so (NUMBL^Val88Met^; gnomAD = 4), were predicted to impair NUMB and NUMBL regulatory functions (43, 58, 59). This prediction was tested by analyzing the effect of the respective NUMB/NUMBL mutations on Notch1 expression and function. Transgenic expression of the respective mutant protein in CRISPR/Cas9-generated NUMB/NUMBL-deficient human embryonic kidney 293 (HEK293) cells revealed that their expression was similar to that of WT NUMB (NUMB^WT^) and NUMBL (NUMBL^WT^) proteins (Figure 4, B and C). However, whereas transgenic NUMB^WT^ and NUMBL^WT^ decreased Notch1 expression in HEK293 cells, the NUMB^Leu94Phe^, NUMBL^Ser79IIe^, and NUMBL^Val88Met^ mutants failed to do so. Similarly, transgenic NUMB^WT^, and to a lesser extent NUMBL^WT^, decreased nuclear N1c expression, whereas the mutant proteins failed to do so (Figure 4, D and E). Cotransfection studies revealed that NUMB^Leu94Phe^ behaved as a dominant-negative mutation that suppressed the capacity of NUMB^WT^ to decrease Notch1 and Notch1c expression (Figure 4D). NUMBL^Ser79IIe^ and NUMBL^Val88Met^ also behaved as dominant-negative mutants by antagonizing the decrease in N1c expression induced by NUMBL^WT^ (Figure 4E). Studies in PBMCs from patients with the NUMB or NUMBL mutations showed upregulation of Notch1 expression on the Tregs of all 3 patients (Supplemental Figure 6, A–C). Expression of N1c reflected the functional impact of these mutations, as revealed in the vitro testing (Figure 4, D and E), with the different mutations falling along a spectrum of NUMB^Leu94Phe^ > NUMBL^Ser79IIe^ > NUMBL^Val88Met^ (Supplemental Figure 6, A–F). These results established the fact that the identified NUMB/NUMBL mutations were functionally deleterious and that patients with MIS-C may harbor mutations in the Notch pathway that contribute to disease pathogenesis.

### Poly I:C–induced multiorgan inflammatory disease in Foxp3^EGFPCre^ R26^N1c/+^ mice.

**Figure 5:**
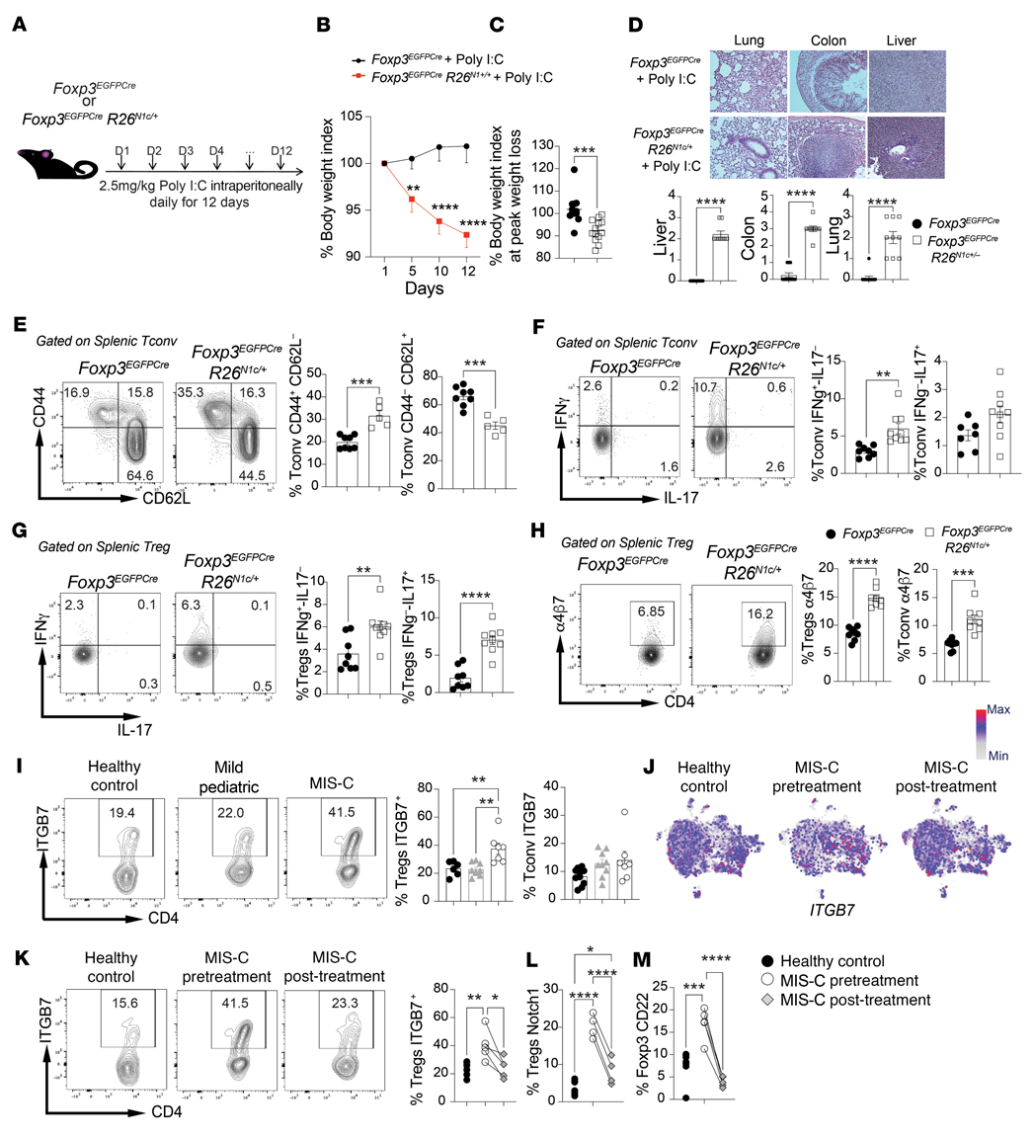
Poly I:C–induced multiorgan inflammatory disease in *Foxp3*^EGFPCre^ *R26*^N1c/+^ mice. (**A**) Experimental scheme. Mice were injected i.p. with poly I:C daily for 12 days. (**B** and **C**) Weight indices of *Foxp3^EGFPCre^* and *Foxp3^EGFPCre^* *R26*^N1c/+^ mice subjected to poly I:C treatment. (**D**) H&E-stained sections and inflammation score for liver, gut, and lung tissues isolated from mice in the indicated groups (original magnification, ×200). (**E**) Flow cytometric analysis and graphical representation of naive (CD4^+^CD44^–^CD62L^+^) and activated (CD4^+^CD44^+^CD62L^–^) Tconv cells. (**F** and **G**) Flow cytometric analysis and graphical representation of IFN-γ and IL-17 expression in Tconv cells (**F**) and Tregs (**G**) in the respective poly I:C–treated mouse groups. (**H**) Flow cytometric analysis and graphical representation of α4β7 expression in Tregs and Tconv cells from mice in the indicated groups. (**I**) Flow cytometric analysis and graphical representation of α4β7 expression in Tregs and Tconv cells from individuals in the indicated groups. (**J**) Relative expression of *ITGB7* in the different clusters inferred from scRNA-Seq data. Max, maximum; Min, minimum. (**K**) Flow cytometric analysis and cell frequencies of α4β7 (ITGB7) expression on circulating CD4^+^FOXP3^+^ Tregs in healthy controls and patients with MIS-C before and after treatment. (**L** and **M**) Frequencies of cells expressing Notch1 (**L**) and CD22 (**M**) on circulating CD4^+^FOXP3^+^ Tregs from healthy controls and patients with MIS-C before and after treatment. Each symbol represents 1 mouse (**B**–**I**), 1 cell (**J**), or 1 human (**I** and **K**–**M**). Numbers in the flow plots indicate percentages. Error bars indicate the SEM. *P < 0.05, **P < 0.01, ***P < 0.001, and ****P < 0.0001, by 2-way ANOVA with Šidák’s post hoc analysis (**B**), Student’s *t* test (**C** and **D**), and 1-way ANOVA with Dunnett’s post hoc analysis (**E**–**I**, and **K**–**M**).

To further delineate the mechanisms by which increased Notch1 signaling in CD4^+^ T cells promotes MIS-C, and in view of the critical role played by Tregs in licensing Notch1-dependent immune dysregulation (41, 42), we used a mouse model in which the intracellular domain of Notch1 (N1c) was conditionally expressed from the Rosa26 locus (*R26*^N1c/+^) in Tregs, using a *Foxp3* promoter–regulated Cre recombinase fused with EGFP (*Foxp3*^EGFPCre^) (Figure 5A) (41). Treatment of *Foxp3*^EGFPCre^ *R26*^N1c/+^ mice with poly I:C, a proxy model of infection with RNA viruses (36, 60–62), resulted in progressive weight loss and multiorgan inflammation. In contrast, poly I:C–treated control *Foxp3*^EGFPCre^ mice were minimally affected (Figure 5, B–D). Analysis of CD4^+^ T cells of *Foxp3*^EGFPCre^*R26*^N1c/+^ mice revealed that their activation phenotype recapitulated that of CD4^+^ T cells of patients with MIS-C, including increased memory markers (CD44^+^CD62L^–^) and heightened IFN-γ production by both Tregs and Tconv cells (Figure 5, E–G).

Most patients with MIS-C present with GI symptoms (Supplemental Table 1) (13, 14). Notably, the Tregs, and to a lesser extent the Tconv cells, of the *Foxp3*^EGFPCre^*R26*^N1c/+^ mice had increased expression of the gut-homing integrin α4β7 (Figure 5H). Increased expression of integrin β7 (ITGB7) was also observed on the circulating Tregs of patients with MIS-C, but not on those of pediatric patients with acute COVID-19, in agreement with a critical role of Notch1 in driving the expression of this marker (Figure 5I). Consistent with this finding, patients with MIS-C exhibited an increase in CD62L^–^CD38^+^ mucosally imprinted Tregs (Supplemental Figure 7A) (63, 64). scRNA-Seq analysis revealed increased expression of *ITGB7* transcripts (Figure 5J and Supplemental Figure 7B). Expression of integrin α4β7 on Tregs from patients with MIS-C declined after treatment, in synchrony with decreased Notch1 and CD22 expression (Figure 5, K–M). These results indicated that increased Notch1 activity in Tregs predisposes to multiorgan inflammation in the context of a viral trigger and promotes Treg gut homing.

### Notch1-mediated CD22 upregulation on Tregs promotes multiorgan inflammation.

**Figure 6:**
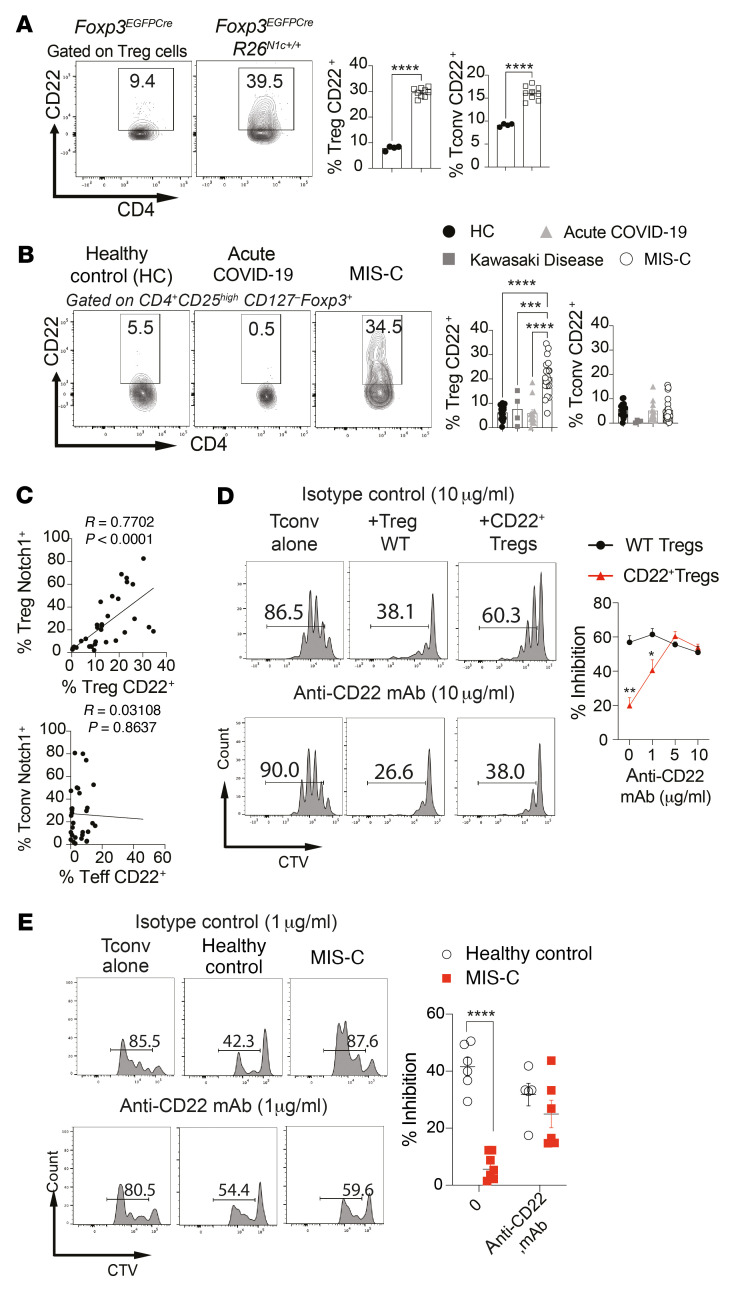
Notch1 destabilizes Treg-suppressive function by a CD22-dependent mechanism. (**A**) Flow cytometric analysis and graphical representation of CD22 expression on splenic Tregs and Tconv cells of poly I:C–treated *Foxp3*^EGFPCre^ and *Foxp3*^EGFPCre^ *R26*^N1c/+^ mice. (**B**) Flow cytometric analysis and cell frequencies of CD22 expression on circulating CD4^+^FOXP3^+^ Treg and CD4^+^FOXP3^–^ Tconv cells from healthy controls and patients with either mild pediatric COVID or MIS-C. (**C**) Correlation analysis of CD22 expression on Tregs and Tconv cells of patients with MIS-C and controls as a function of Notch1 expression on these cells. (**D**) In vitro suppression of Tconv cell proliferation by *Foxp3*^EGFPCre^ and CD22^+^ *Foxp3*^EGFPCre^ *R26*^N1c/+^ Tregs in the presence of increasing concentrations of anti-CD22 mAb. (**E**) In vitro suppression of human Tconv cell proliferation by Tregs isolated from healthy controls or patients with MIS-C in the absence of presence of anti-CD22 mAb. CTV, Cell Trace Violet. Each symbol represents 1 mouse (**A**) or 1 human (**B** and **C**). Numbers in flow plots indicate percentages. Error bars indicate the SEM. *P < 0.05, **P < 0.01, ***P < 0.001, and ****P < 0.0001, by Student’s *t* test (**A**), 1-way ANOVA with Dunnett’s post hoc analysis (**B**), 2-way ANOVA with Sidak’s post hoc analysis (**D** and **E**), and Pearson’s correlation analysis (**C**).

To delineate the mechanisms by which Notch1 signaling in Tregs promotes multiorgan inflammation in the context of a viral trigger, we analyzed the transcriptome of Notch1c-expressing Tregs for pathways involved in the immune dysregulation (41). We found upregulation of CD22, a member of the Siglec family of lectins normally found in B cells, where it acts to regulate B cell receptor signaling (45). In particular, CD22 directs B cells to the intestinal lymphoid and mucosal tissues by upregulating expression of the gut-homing receptor α4β7 (44). Flow cytometric analysis of Tregs from *Foxp3^EGFPCre^* *R26^N1c/+^* mice revealed increased CD22 expression upon treatment of the mice with poly I:C (Figure 6A). Expression of CD22 in Tregs was abrogated upon Treg-specific deletion of *Rbpj*, the gene encoding the Notch canonical pathway transcriptional cofactor RBPJ (Supplemental Figure 8A). Analysis of peripheral blood Tregs from patients with MIS-C revealed increased expression of CD22 that strongly correlated with Notch1 expression on these cells (Figure 6B). CD22 expression was also increased in patients with NUMB/NUMBL mutations, in accord with the impact of the mutation (Supplemental Figure 6, G–I). In contrast, CD22 was minimally expressed on CD4^+^ Tconv cells of control individuals, patients with acute COVID-19, and patients with MIS-C, and it did not correlate with Notch1 expression in these cells (Figure 6, B and C). These results indicated that CD22 was upregulated on Tregs with active Notch1 signaling.

We analyzed the functional relevance of CD22 expression on Tregs by performing in vitro Treg suppression assays on Tregs pooled from different mice, which revealed profoundly defective suppressive function of CD22^+^ Tregs from *Foxp3^EGFPCre^* *R26^N1c/+^* mice compared with Tregs from *Foxp3*^EGFPCre^ control mice. This defect was corrected upon treatment of Tregs with anti-CD22 mAb (Figure 6D). Similarly, the suppressive function of Tregs from patients with MIS-C Tregs was profoundly deficient compared with that of Tregs from healthy controls, which was corrected upon treatment of the cells with anti-CD22 mAb (Figure 6E).

**Figure 7:**
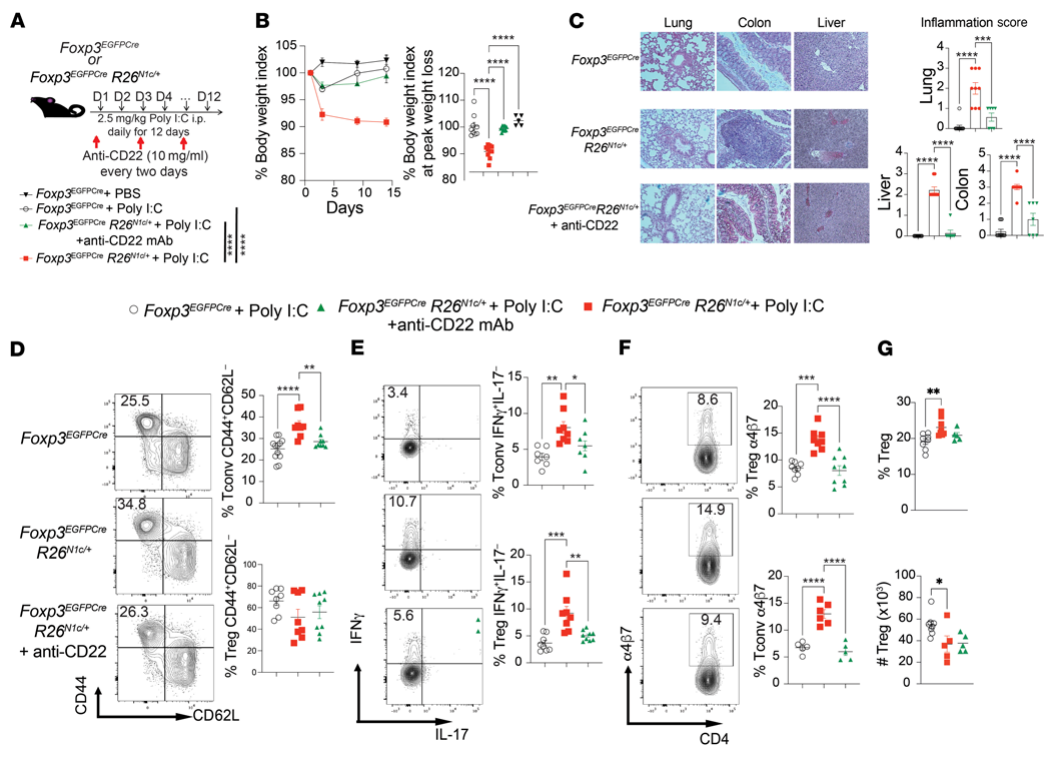
A Treg Notch1/CD22 axis promotes multiorgan inflammation. (**A**) Experimental scheme. Mice were injected i.p. with poly I:C daily for 12 days. (**B**) Weight indices of *Foxp3*^EGFPCre^ and *Foxp3*^EGFPCre^ *R26*^N1c/+^ mice subjected to poly I:C treatment. (**C**) H&E-stained sections and inflammation score of liver, gut, and lung tissues isolated from the indicated mouse groups (original magnification, ×200). (**D**) Flow cytometric analysis and graphical representation of naive (CD4^+^CD44^–^CD62L^+^) and activated (CD4^+^CD44^+^CD62L^–^) Tconv cells. (**E**) Flow cytometric analysis and graphical representation of IFN-γ and IL-17 expression in Tconv cells (**E**) and Tregs (**F**) from mice in the respective poly I:C treatment groups. (**F**) Flow cytometric analysis and graphical representation of α4β7 expression in Tregs and Tconv cells from mice in the indicated groups. (**G**) Frequencies and number of splenic Tregs in the respective groups. Each symbol represents 1 mouse (**B**–**H**). Numbers in the flow plots indicate percentages. Error bars indicate the SEM. *P < 0.05, **P < 0.01, ***P < 0.001, and ****P < 0.0001, by 2-way ANOVA with Šidák’s post hoc analysis (**B**) and 1-way ANOVA with Dunnett’s post hoc analysis (**B**–**G**).

To determine the role of CD22 expression on Tregs in the multiorgan inflammatory disease triggered by poly I:C treatment of *Foxp3^EGFPCre^* *R26^N1c/+^* mice, we examined the effect of therapy with a neutralizing anti-CD22 mAb on disease outcomes in these mice. Anti-CD22 mAb treatment prevented the weight loss and multiorgan inflammation induced by poly I:C treatment (Figure 7, A–C). Treatment with this mAb downregulated the activation of splenic CD44^+^CD62L^–^ Tconv cells and the expression of IFN-γ by Tregs and Tconv cells (Figure 7, C–F). Anti-CD22 mAb treatment also downregulated the expression of α4β7 by splenic Tregs (Figure 7F). Anti-CD22 mAb treatment did not deplete Tregs (Figure 7G). In contrast, B cell depletion with an anti–B cell–specific anti-CD20 mAb failed on its own to protect against disease or to abrogate protection by anti-CD22 mAb treatment (Supplemental Figure 8, B and C). Analysis of gut lamina propria lymphocytes (LPLs) revealed increased infiltration with activated (CD44^+^CD62L^–^) Tconv cells and Tregs, with increased expression of IFN-γ that was similarly downregulated upon treatment with anti-CD22 mAb (Supplemental Figure 8, D and E). Overall, these results indicated that anti-CD22 mAb treatment suppressed both the gut and systemic inflammation induced by poly I:C treatment of *Foxp3*^EGFPCre^ *R26*^N1c/+^ mice.

To further link the above results with our human studies, we used another mouse model in which a floxed *Numb* allele was conditionally deleted in Tregs (*Foxp3*^YFPCre^ *NUMB*^Δ/Δ^) (Supplemental Figure 9, A and B). Treatment of *Foxp3*^YFPCre^ *NUMB*^Δ/Δ^ mice with poly I:C i.p. resulted in progressive weight loss similar to what was observed in poly I:C–treated *Foxp3*^EGFPCre^ *R26*^N1c/+^ mice. In contrast, poly I:C treatment of control *Foxp3*^YFPCre^ had no effect (Supplemental Figure 9, C and D). Analysis of CD4^+^ T cells from *Foxp3*^YFPCre^ *NUMB*^Δ/Δ^ mice revealed that their activation phenotype recapitulated that of CD4^+^ T cells from patients with MIS-C, including increased memory markers (CD44^+^CD62L^–^) and heightened IFN-γ production by Tconv cells (Supplemental Figure 9, E and F). Moreover, Tregs from poly I:C–treated *Foxp3*^YFPCre^ *NUMB*^Δ/Δ^ mice also showed an upregulation of Notch1, N1c, CD22, and α4β7 that recapitulated the Treg phenotype of patients with MIS-C and poly I:C–treated *Foxp3*^EGFPCre^ *R26*^N1c/+^ mice (Supplemental Figure 9, G–J). Finally, treatment of *Foxp3*^YFPCre^*NUMB*^Δ/Δ^ mice with an anti-CD22 mAb prevented disease development following poly I:C treatment, downregulated Notch pathway and gut-homing markers, and suppressed CD4^+^ T cell expansion and activation and IFN-γ expression in both the spleen and gut (Supplemental Figure 9, A–M). Overall, these results indicated that Notch1-dependent induction of CD22 expression on Tregs played a crucial proinflammatory role in poly I:C–treated *Foxp3*^EGFPCre^ *R26*^N1c/+^ mice.

**Figure 8:**
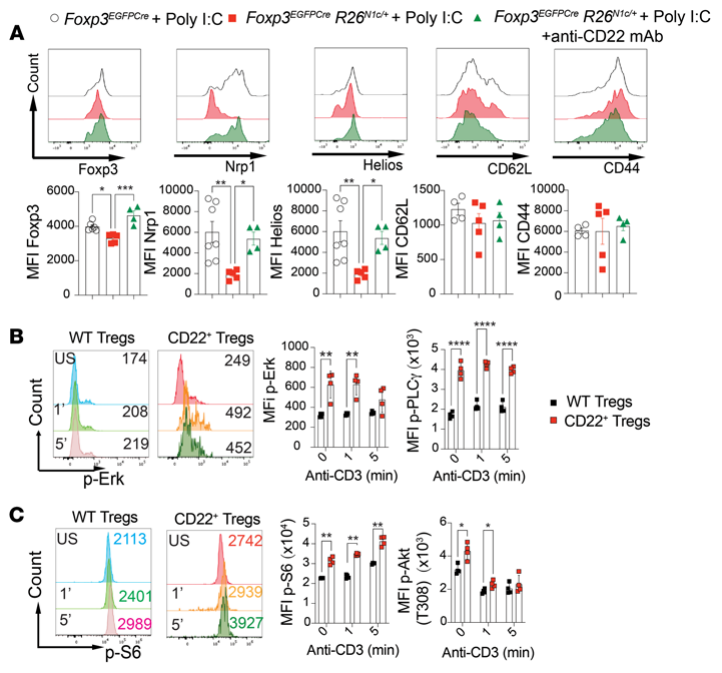
CD22 augments TCR signaling in Tregs. (**A**) Flow cytometric analysis and MFI of colonic markers of Tregs from poly I:C–treated *Foxp3*^EGFPCre^ and *Foxp3*^EGFPCre^*R26*^N1c/+^ mice cotreated with isotype a control mAb or an anti-CD22 mAb. (**B**) Flow cytometric analysis and MFI of p-Erk and p–PLC-γ expression induced by anti-CD3 mAb treatment of *Foxp3*^EGFPCre^ and CD22^+^ *Foxp3*^EGFPCre^*R26*^N1c/+^ Tregs. (**C**) Flow cytometric analysis and MFI of p-S6 and p-AKT (T308) expression induced by anti-CD3 mAb treatment of *Foxp3*^EGFPCre^ and CD22^+^ *Foxp3*^EGFPCre^*R26*^N1c/+^ Tregs. Numbers in the flow plots indicate percentages or MFI. Each symbol represents 1 mouse (**A**–**C**). Error bars indicate the SEM. *P < 0.05, **P < 0.01, ***P < 0.001, and ****P < 0.0001, by 1-way ANOVA with Dunnett’s post hoc analysis (**A**) and 2-way ANOVA with Šidák’s post hoc analysis (**B** and **C**).

To determine the mechanisms by which CD22 subverted Treg function, we analyzed the steady-state transcriptome of a pool of CD22^+^ Tregs from *Foxp3*^EGFPCre^ *R26*^N1c/+^ mice compared with control *Foxp3*^EGFPCre^ Tregs or CD22^–^ Tregs from *Foxp3*^EGFPCre^ *R26*^N1c/+^. KEGG and GO pathway analyses showed increased expression of genes involved in the regulation of the immune response, T cell migration, and Notch signaling (Supplemental Figure 10, A–F). Furthermore, we analyzed by flow cytometry the phenotypes of CD22^+^ colonic and splenic Tregs from *Foxp3*^EGFPCre^ *R26*^N1c/+^ mice isolated at steady state and following poly I:C treatment compared with colonic and splenic Tregs from similarly treated *Foxp3*^EGFPCre^ mice. The CD22^+^ Tregs exhibited decreased expression of Helios and NRP-1 both at steady state and after poly I:C treatment in the face of similar expression levels of markers of T cell activation including CD44, indicating their decreased stability (Supplemental Figure 10, G and H). In agreement with this conclusion, Foxp3 expression also decreased in CD22^+^ Tregs following poly I:C treatment. Treatment with an anti-CD22 mAb reversed those defects in both colonic and splenic Tregs (Figure 8A and Supplemental Figure 10, H and I).

**Figure 9:**
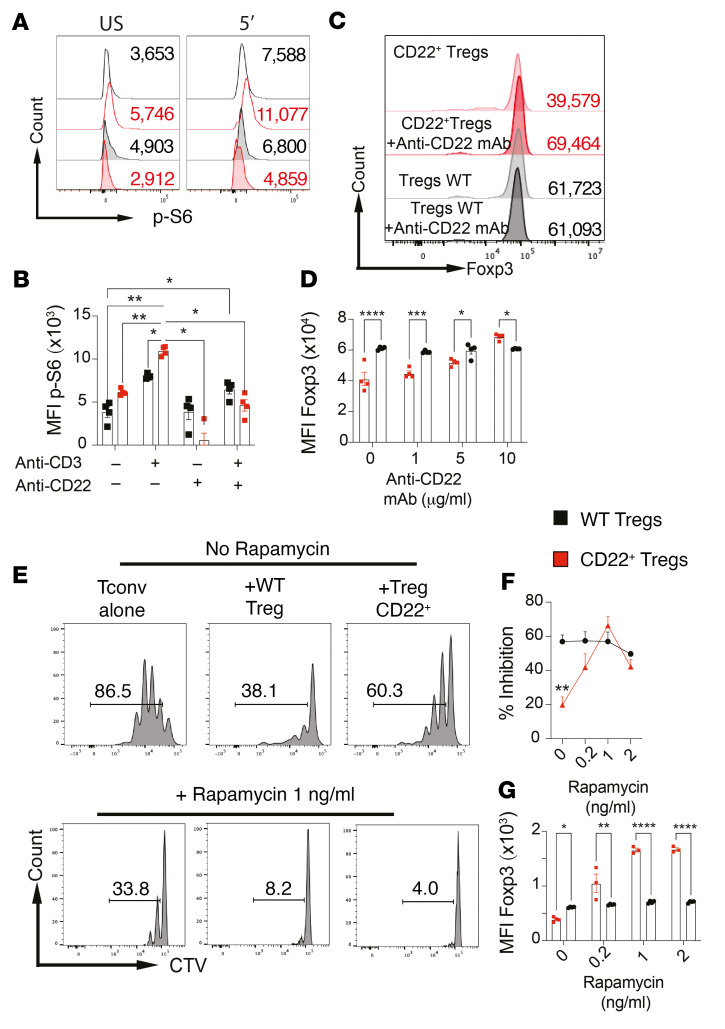
CD22 destabilizes Tregs by an mTOR-dependent mechanism. (**A** and **B**) Flow cytometric analysis (**A**) and MFI (**B**) of p-S6 expression induced by anti-CD3 mAb treatment of *Foxp3*^EGFPCre^ and CD22^+^ *Foxp3*^EGFPCre^*R26*^N1c/+^ Tregs that were treated or not with anti-CD22 mAb. (**C** and **D**) Foxp3 MFI in Tregs from in vitro suppression of Tconv cell proliferation by *Foxp3*^EGFPCre^ and CD22^+^ *Foxp3*^EGFPCre^*R26*^N1c/+^ Tregs in the presence of increasing concentrations of anti-CD22 mAb. (**E** and **F**) In vitro suppression of Tconv cell proliferation by *Foxp3*^EGFPCre^ and CD22^+^ *Foxp3*^EGFPCre^*R26*^N1c/+^ Tregs in the presence of increasing concentrations of rapamycin. (**G**) Foxp3^+^ MFI in Tregs from in vitro suppression of Tconv cell proliferation by *Foxp3*^EGFPCre^ and CD22^+^ *Foxp3*^EGFPCre^*R26*^N1c/+^ Tregs in the presence of increasing concentrations of rapamycin. Numbers in flow plots indicate percentages or MFI. Each symbol represents 1 mouse (**A**–**G**). Error bars indicate the SEM. *P < 0.05, **P < 0.01, ***P < 0.001, and ****P < 0.0001, by 2-way ANOVA with Šidák’s post hoc analysis (**B**, **D**, **F**, and **G**).

CD22 regulates B cell receptor signaling by forming a molecular scaffold that enables coordinated docking of different downstream signaling pathways (65). Analysis of CD22^+^ Tregs revealed enhanced activation of T cell receptor–coupled pathways compared with control Tregs, with increased phosphorylation of ERKs and phospholipase C γ 1 (pPLCγ1) (Figure 8B). Downstream of the PI3/kinase pathway, expression levels of phosphorylated AKT (p-AKT) kinase at residue T308, a target of upstream phosphoinositide-dependent kinases, and the mTOR complex 1 (mTORC1) substrate S6 kinase were also increased (Figure 8C) (66, 67). Treatment with an anti-CD22 mAb downregulated S6 phosphorylation, thus indicating active intracellular signaling by CD22 in Tregs (Figure 9, A and B). Anti-CD22 mAb treatment corrected the decreased MFI of Foxp3 found at the end of the in vitro Treg-suppressive assay, indicative of reversal of CD22^+^ Treg instability (Figure 9, C and D). Treatment with the mTOR inhibitor rapamycin reversed the regulatory defect in CD22^+^ Tregs and the associated loss of Foxp3 expression (Figure 9, E–G). These results indicated that CD22 positively enhanced T cell receptor signaling in Tregs, leading to their destabilization and loss of regulatory function by an mTORC1-dependent mechanism.

## Discussion

In this study, we demonstrate that MIS-C entails the mobilization of a Treg-specific pathway involving Notch1/CD22 signaling that promotes immune dysregulation and that can be demonstrated in both humans and proxy mouse models. Patients with MIS-C, but not children or adults with acute COVID-19, demonstrated increased Notch1 expression on circulating CD4^+^ Tregs and Tconv cells, all of which declined precipitously following antiinflammatory therapy. The pathogenic function of this pathway was confirmed by the identification of dominant-negative mutations in PTB domains of *NUMB* and *NUMBL* in patients with MIS-C that resulted in increased Notch1 expression. This observation was also supported by the demonstration of a role for Notch pathway–related mutations in MIS-C using Monte Carlo simulation and Fisher’s exact test. Uniquely, patients with MIS-C exhibited increased CD22 expression on Tregs, but not Tconv cells, which could be demonstrated in mice to involve Notch1 signaling via the RBPJ-k canonical pathway. CD22 blockade was sufficient to inhibit the immune dysregulation triggered by Notch1 signaling in Tregs in the poly I:C proxy viral infection model, highlighting the critical role of this molecule in MIS-C disease pathogenesis.

Our previous studies have shown Notch4 to be specifically upregulated on circulating Tregs in adult patients with COVID-19; their origin could be traced to the lung in mouse models of viral infection (36). Notch4 was similarly upregulated on circulating Tregs of pediatric patients with acute COVID-19, while both Notch4 and Notch1 were upregulated on those of patients with MIS-C. However, expression of Notch1 and Notch4 on MIS-C Tregs was mutually exclusive, suggesting that the respective Treg populations were ontogenically distinct. These findings suggest that Notch4 and Notch1 regulate distinct checkpoints in the evolution of immune dysregulation following SARS-CoV-2 and other viral infections. Thus, Notch4 appears critical for licensing lung inflammation following SARS-CoV2, influenza, and related viral infections (36). In contrast, increased Notch1 expression on Tregs and Tconv cells, with at least some of the latter being derived from destabilized Notch1^+^ Tregs, may favor the evolution of Th1-skewed systemic inflammation (41, 42). Overall, our studies provide a mechanistic framework for the evolution of autoimmunity in MIS-C by establishing Notch pathway–dependent Treg dysfunction as a critical step in this process (16, 17, 25)

Mouse studies revealed that a critical step by which Notch1 signaling in Tregs promotes systemic inflammation involves its induction of CD22, an inhibitory receptor previously associated with B cells, where it functions as a regulator of B cell receptor signaling. More recently, CD22 has been described as directing the homing of B cells to the gut lymphoid and mucosal tissues by virtue of its upregulation of the gut-homing integrin α4β7 (44). Consistent with these findings, treatment of mice whose Tregs expressed a gain-of-function Notch1 mutant with an anti-CD22–blocking antibody rescued their gut and systemic inflammation following treatment with poly I:C. CD22 impaired the in vitro Treg-suppressive function, an effect that was reversed by treatment with the anti-CD22–blocking antibody. Notwithstanding its function as an inhibitory receptor, Tregs expressing CD22 demonstrated increased T cell receptor signaling with increased mTORC1 activity, leading to a defective Treg-suppressive function that was reversed by treatment with the mTOR inhibitor rapamycin.

MIS-C is a rare complication of SARS-CoV-2 infection (68), suggesting a genetic predisposition to this disorder. In that regard, mutations in a number of immune-regulatory genes have been described in MIS-C, including *SOCS1*, *XIAP*, and *CYBB* as well as HLA class I alleles and rare heterozygous mutations in genes associated with hemophagocytic lymphohistiocytosis (25, 27, 32, 55). We found variants in a number of Notch pathway genes in patients with MIS-C. Importantly, dominant-negative loss-of-function mutations in *NUMB* and *NUMBL* found in patients with MIS-C resulted in increased Notch1 expression and Notch1 signaling, consistent with the pathogenic function of these mutations in this pathway in promoting MIS-C in the context of a systemic viral infection such as with SARS-CoV2.

Collectively, these results allow for the construction of a model that traces the evolution of MIS-C. An initial infection with the SARS-CoV2 virus resulted in the expansion of Notch4 and Notch1 Tregs, the latter favored by rare genetic variants found in the susceptible host. A subset of Notch1^+^ Tregs upregulated CD22, which severely impaired their regulatory function and drove their homing to the gut, where they promoted inflammation, possibly instigated by the persistence of SARS-CoV2 in the GI tract and/or in reaction to the gut microbiota (14). This cascading immune-dysregulatory process may be amplified by heightened responses of some children to pathogen- and damage-associated signals (21), which aggravates the systemic spread of inflammation and the broad disruption of tissue Treg function. Importantly, Notch-related immune dysregulation may extend to involve other disease states associated with inflammation including other viral infections, inflammatory bowel disease, and graft-versus-host disease (41, 42, 44, 69). This dysregulation was reversible by antiinflammatory therapy that targeted cytokines involved in Notch1 induction. Our results also suggest that blockade of CD22 or treatment with the mTORC1/-2 inhibitor rapamycin may provide an alternative therapy for those patients who prove resistant to standard-of-care antiinflammatory therapy.

## Methods

### Patient cohorts contributing to the flow cytometric, transcriptomic, and functional studies

#### Patients with MIS-C and pediatric patients with COVID-19.

Peripheral blood samples were obtained from pediatric patients with COVID-19 (*n* = 9) and patients with MIS-C (*n* = 23), who were prospectively recruited from Boston Children’s Hospital (BCH) as part of the Taking on COVID-19 Together Study and the KD Biorepository, between May 2020 and April 2021. In addition, blood samples were also collected from children admitted to the Marmara University Hospital in Istanbul, Turkey (*n* = 8 COVID-19 patients, *n* = 8 MIS-C patients from December 2020 to January 2021); Istanbul University-Cerrahpaşa in Istanbul, Turkey (*n* = 20 COVID-19 patients, *n* = 2 MIS-C patients from July 2020 to January 2021); the Gaslini Institute in Genoa, Italy (*n* = 4 MIS-C patients from December 2020 to March 2021); and the Bambino Gesù Children’s Hospital in Rome, Italy (*n* = 13 COVID-19 patients, *n* = 12 MIS-C patients from March 2020 to April 2021). The clinical characteristics of the patients with MIS-C and pediatric patients with COVID-19 are reported in Supplemental Table 1. All patients with MIS-C met the CDC case definition for MIS-C (46). Patients requiring ICU admission and/or vasopressor support, or those who developed coronary artery aneurysms (*z* score ≥2.5) were classified as having severe MIS-C.

Children with COVID-19 presented with either a fever, respiratory illness, and/or known COVID-19 exposure and were found to be positive for SARS-CoV-2 by PCR test. Moderate pediatric COVID-19 was defined by a supplemental oxygen requirement and care on the pediatric ward, whereas severe disease required ICU admission and/or bilevel positive airway pressure (BiPAP) or mechanical ventilation. All other children with COVID-19 were defined as having mild disease.

#### Adult patients with COVID-19.

Twelve patients, who were previously described (36), were included in this study.

#### KD.

Five children with KD provided peripheral blood samples through the KD Biorepository at BCH from October 2020 to January 2021 (Supplemental Table 1). These patients fulfilled the clinical criteria for either complete or incomplete KD, as outlined by the American Heart Association (AHA) (70). Furthermore, all of these patients tested negative for SARS-CoV-2 by both PCR and serology and had no known close contacts with individuals with COVID-19.

#### Controls.

Peripheral blood samples were obtained from pediatric control participants recruited from BCH (*n* = 6), Istanbul University-Cerrahpaşa (*n* = 7), and the Gaslini Institute (*n* = 5). Clinical characteristics of the control study participants can be found in Supplemental Table 1.

#### Sample processing.

Peripheral blood samples were obtained at study enrollment in either sodium heparin or EDTA tubes. At each site, PBMCs were isolated by Ficoll density-gradient centrifugation and cryopreserved in liquid nitrogen.

### Gene pathway analyses using Fisher’s exact and Monte Carlo tests

Whole-genome or whole-exome sequences were obtained from patients with MIS-C and pediatric patients with COVID-19. These patients were recruited from the Taking on COVID-19 Together Study at BCH (*n* = 30 with MIS-C; *n* = 21 with COVID-19) as well as from Marmara University Hospital (*n* = 9 with MIS-C; *n* = 3 with COVID-19).

In addition, whole-exome sequencing (WES) and whole-genome sequencing data were obtained from 1,885 families (4,682 samples) at the Manton Center for Orphan Disease Research and were used for gene enrichment tests (Manton Center for Orphan Disease Research: https://www.mantonfoundation.org/). Data were divided into 4 groups depending on the following phenotypes: obesity (*n* = 86 samples), myopathy (*n* = 310 samples), autism-ADHD (*n* = 1,296 samples), or rare diseases (*n* = 2,990 samples). Rare diseases were subdivided depending on the sequencing provider: Broad Institute (*n* = 1,006 samples), GeneDx (*n* = 715 samples), BCH (*n* = 545 samples), and others (*n* = 724 samples). Samples from 162 families (*n* = 385 samples) with immunodeficiency, allergic dysregulation, autoimmunity, or recurrent infections obtained from BCH repositories were also included in our analysis.

### Patient screening for Notch-related genes

Blood samples from patients with MIS-C enrolled in the Overcoming COVID-19 Immunobiology Study were obtained from 20 large pediatric sites in the United States and sent for WES (*n* = 88 MIS-C patients from June 2020 to May 2021). DNA was isolated from whole blood using the Gentra Puregene Blood Kit (QIAGEN) or by GeneDx using IDT xGen probes. Libraries for WES were prepared using the SureSelectXT2 *Homo sapiens* All Exon V6 Kit (Agilent Technologies). Paired-end sequencing was performed with an Illumina HiSeq- 2000, generating 150 base reads. Sequencing alignment to the hg19/GRCh37 reference build was performed using the Burrows-Wheeler Aligner (71). Variant calling and candidate variant analysis were completed using the BCH Genomic Learning System, as previously described (53). Minor allelic frequencies for the specified variants were identified using the Genome Aggregation Database (72).

### Mice

The *Foxp3^EGFPCre^* [B6129S-Tg (Foxp3-EGFP/iCre)1aJbs/J] and *Rosa26^N1c/+^* mouse strains were obtained from The Jackson Laboratory. *Rbpj1^fl/fl^* (B6.129P2-*Rbpj^tm1Hon^* HonRbrc) mice were a gift of Tasuku Honjo (Kyoto University, Kyoto, Japan).

### scRNA-Seq

Cryopreserved PBMCs were thawed in plain RPMI (HyClone) prewarmed to 37°C, washed in PBS (HyClone), and resuspended in FACS buffer (PBS with 1.5% FBS [Genesee Scientific]) and 2.5 mM EDTA (Invitrogen, Thermo Fisher Scientific) for CD4^+^ T cell enrichment through negative selection (Miltenyi Biotec). Samples were studied in 2 independent experiments: experiment 1 included 3 pediatric controls, 1 pretreatment MIS-C patient, and 4 post-treatment MIS-C patients; experiment 2 included 1 pediatric control, 2 pretreatment MIS-C patients, and 1 post-treatment MIS-C patient. Detailed procedures are described in Supplemental Methods.

### scRNA-Seq clustering analyses

Sequencing data from each 10x run were processed with the CellRanger pipeline (10x Genomics) for demultiplexing and gene alignment (73). The resulting raw count matrices were imported in R (version 4.0.2 and above) using Seurat (version 4.0.3) (50). Data from all 3 runs were merged into 1 Seurat object. Genes detected in fewer than 1 per 10,000 cells were filtered out, leaving a transcriptomic coverage of 21,675 genes. High-quality cells with more than 1,400 unique molecular identifiers (UMIs), more than 700 genes, a log_10_(gene) to log_10_(UMI) ratio of greater than 0.84, and a mitochondrial to nuclear gene ratio of less than 0.08 were retained for downstream analyses. Quality control revealed no significant batch effect, and similar distributions were observed for the metrics mentioned above across different runs and experiments. Detailed procedures are described in Supplemental Methods.

### Pseudobulk differential expression analyses

For pseudobulk DEA, gene expression level data were aggregated at the patient level for each subset of interest, namely Tregs and activated Tconv cells. For this analysis, we considered a Treg any cell assigned to Seurat cluster 15 (*FOXP3*-expressing cells) or was annotated as a Treg by Azimuth, which added up to 1,925 Tregs across all 12 patients. Similarly, we considered an activated Tconv cell any cell assigned to Seurat clusters 9–14 and annotated as CD4 T central memory (Tcm) cells, CD4 T effector memory (Tem) cells, CD4 cytotoxic T lymphocytes (CTLs), or proliferating CD4^+^ T cells by Azimuth (*n* = 6,674 cells). Detailed procedures are described in the Supplemental Methods.

### Gene pathway analysis using Fisher’s exact and Monte Carlo tests

To determine whether a pathway was relevant to MIS-C or acute COVID-19 (pediatric patients with mild or severe disease), a comparison between MIS-C or acute-COV19 and the 8 databanks described above was performed as detailed in Supplemental Methods.

### CRISPR/Cas9-knockout generation

HEK293 cells were transfected with the CRISPR/Cas9-knockout plasmids NUMB/NUMBL (Santa Cruz Biotechnology) using a CalPhos Mammalian Transfection kit (Takara) following the manufacturer’s protocol. CRISPR^+^ cells were isolated by FACS and plated at a very low density to induce the formation of clonal colonies. Individual clones were then isolated and tested for knockout efficiency by flow cytometry.

### In vitro Notch1 induction

Human Tregs from healthy donors were isolated by cell sorting (Sony Sorter, MA900) based on CD3, CD4, CD25^hi^, and CD127^lo^ expression. Tregs were seeded at 1 × 10^4^ cells in 96-well plates and then stimulated with CD3/CD28 Dynabeads (Thermo Fisher Scientific) alone or in the presence of recombinant IL-1β, IP-10, IL-6, IFN-γ, and IFN-λ2 (10 μg /mL; Peprotech) for 72 hours. Notch1 expression on Foxp3^+^ Tregs was then assessed by flow cytometry.

### NUMB and NUMBL mutagenesis and cell transfection

pCMV6-AC-Numb-GFP plasmids encoding for human NUMB (NM_001005745) or human NUMBL (NM_004756) were purchased from OriGene (RG209744). The directed NUMB (c.280 C>T, p.Leu 94 Phe) and NUMBL (c.236G>T, p.Ser79Ile; c.262G>A p.Val88Met) mutagenesis plasmids were generated with standard cloning techniques using pCMV6-AC-NUMB-GFP as a template. NUMB/NUMBL-deficient HEK293T cells were cultured in 10% FBS DMEM (Gibco, Thermo Fisher Scientific) supplemented with 100 U/mL penicillin and 100 μg/mL streptomycin (Invitrogen, Thermo Fisher Scientific). The day before transfection, the NUMB/NUMBL-deficient HEK293 cells were seeded at a density of 5 × 10^5^ cells per well of 6-well plates. The next day, HEK293 cells were transiently transfected with 2 μg of the respective plasmids encoding either WT or mutant NUMB or NUMBL using GeneJuice Transfection Reagent (Merck Millipore) according to the manufacturer’s instructions. After 48 hours of transfection, cells were collected for flow cytometric analysis.

### Cytokine measurements

IL-1β, IL-6, IL-8, IFN-α, IFN-β, IFN-γ, IFN-λ, CXCL10, and TNF were measured using LegendPlex (BioLegend) following the manufacturer’s protocol.

### Poly I:C mouse model

Mice were treated i.p. with 2.5 mg/kg poly I:C HMW (InvivoGen) every 2 days for 12 consecutive days. The weight of the mice was recorded daily. Mice were euthanized and analyzed on day 13. For blockading, CD22 mice were treated with an anti-CD22 antibody every 2 days for 12 consecutive days (InVivoMAb anti–mouse CD22; clone Cy34.1, Bio X Cell). A 20 μg dose of antibody in PBS was given in a final volume of 100 μL, or an isotype control mAb (clone MOPC-21; Bio X Cell) was given. For the CD20 depletion experiment, the mice were administered i.p. 10 μg anti-CD20 mAB (clone MB20-11; Bio X Cell) or an isotype control mAb (clone MOPC-21; Bio X Cell) every 2 days, 6 days before the start of the experiment.

### Histopathology staining

Paraffin-embedded lung, colon and liver sections were stained with H&E or periodic acid–Schiff (PAS) staining. The lung, colon, and liver pathology was scored in a blinded manner, as described previously (74).

### Flow cytometric analysis of mouse and human cells

Antibodies against murine and human antigens used for flow cytometric analyses are described in Supplemental Table 2. The specificity and optimal dilution of each antibody were validated by testing on appropriate negative and positive controls. Intracellular cytokine staining was performed as previously described (41). Dead cells were routinely excluded from the analysis based on the eFluor 780 Fixable Viability Dye (1:1,000 dilution; Thermo Fisher Scientific) staining. Stained cells were analyzed on a BD LSR Fortessa cell analyzer (BD Biosciences), and data were processed using FlowJo (Tree Star).

### Transcriptome profiling

Tregs were isolated from either *Foxp3*^EGFPcre^ or Foxp3^EGFPCre^ *Rosa26*^N1c/+^ mice by cell sorting, and their RNA was isolated using a Qiagen RNeasy Mini Kit (QIAGEN). RNA was then converted into double-stranded DNA (dsDNA) using SMART-Seq, version 4, Ultra Low Input RNA kit (Clontech). dsDNA was then fragmented into 200–300 bp sizes using an M220 Focused ultrasonicator (Covaris) and utilized for the construction of libraries for Illumina sequencing using a KAPA Hyper Prep Kit (Kapa Biosystems). Libraries were then quantified using a Qubit dsDNA HS (High Sensitivity) Assay Kit on an Agilent High Sensitivity DNA Bioanalyzer. Detailed procedures are described in Supplemental Methods.

### In vitro suppression assays

Mouse and human Treg suppression assays were performed as described previously (74). Tregs from *Foxp3^EGFPCre^* and Foxp3*^EGFPCre^* *Rosa26^N1c/+^* mice were sorted on the basis of CD4, YFP, and/or CD22 expression and were used as suppressor cells. Responder cells were cocultured with Tregs at a 1:1 ratio and stimulated at 37°C in 5% CO_2_ for 3 days with 2 μg/mL coated anti-CD3 and 1 μg/mL soluble anti-CD28 in the presence of an increased dose of anti-CD22 mAb or rapamycin in 96-well, round-bottomed plates in triplicate. For human Treg suppression assays, Tregs (CD3^+^CD4^+^CD127^–^CD25^hi^) and CD4^+^ Tconv cells (responder) were isolated from PBMCs by cell sorting. Responder cells were cocultured with Tregs at a 1:1 ratio and stimulated at 37°C in 5% CO_2_ for 3 days with 2 μg/mL coated anti-CD3 and 1 μg/mL soluble anti-CD28 in the presence of 1 μg/mL anti-CD22 mAb or 1 ng/mL rapamycin in 96-well, round-bottomed plates in triplicate. The responder cells were then analyzed for CellTrace dye (Thermo Fisher Scientific) dilution by flow cytometry.

### Analysis of TCR signaling by phosphoflow

Total spleen cells from either *Foxp3^EGFPCre^* or *Foxp3^EGFPCre^* *R26^N1c/N1c^* mice were stimulated at 37°C in nonsupplemented RPMI 1640 using preformed complexes of biotinylated anti-CD3 mAb (clone 145-2C11, BD, 30 μg/mL), anti-CD4 mAb (GK1.5, BD, 30 μg/mL), and streptavidin (60 μg/mL) for 1–5 minutes. The reaction was stopped, and cells were permeabilized using Foxp3 transcription factor staining buffer (eBiosciences) and Perm Buffer III (BD Biosciences). Cells were stained with the antibodies described in Supplemental Table 2. The samples were acquired on a BD Fortessa cytometer, and data were analyzed with FlowJo software.

### Statistics

Student’s 2-tailed *t* test, 1- or 2-way ANOVA, repeated-measures 2-way ANOVA with post test analysis, and a log-rank test of groups were used to compare test groups. Linear regression was used for correlation analysis. For human data analysis, summary statistics were calculated using the number (percentage) for binary and categorical data and the mean (SD) or median (IQR) for continuous data, depending on the normality of the distribution. A p value <0.05 was considered statistically significant.

### Study approval

#### Human studies.

Written informed consent (and assent when appropriate) was provided by the study participants or their health care proxy and by at least 1 parent or legal guardian for minor children. The single-center research protocols were approved by the IRBs of BCH (IRB-P00035409 [Taking on COVID-19 Together], X10-01-0308, IRB-P00005723); Marmara University Hospital (224165); Istanbul University-Cerrahpasa (159066); the Gasilini Institute (egione Liguria ImmunoCOVID19” 0012337/20); and the Bambino Gesù Children’s Hospital (2083_OPBG_2020); BCH (P0021163, P00035489, P00035810, IRB-P00004759 and 04-09-113R). BCH serves as the single IRB for the multicenter Overcoming COVID-19 Immunobiology Study (IRB-P00033157), and the IRBs of all enrollment sites reviewed and approved the protocol. The research protocols for the rare disease cohorts were approved by the IRB of the Manton Center samples (10-02-0053).

#### Animal studies.

All animal studies were reviewed and approved by the Office of Animal Care Resources of BCH.

### Data availability

Any data and materials to be shared will be released via a material transfer agreement. Bulk RNA-Seq data sets have been deposited in the NCBI’s Gene Expression Omnibus (GEO) database (GEO GSE186799). The scRNA-seq data has been deposited at the database of Genotypes and Phenotypes (dpGaP), accession number phs003086.v1.p1.

## Author contributions

MB and TAC conceived the project and designed experiments. MB, QC, RB, PC, MW, JF, PSL, TMFC, EZA, EK, EKA, AO, PB, FA, PO, AAKS, HC, MYG, FBO, EAC, YZ, and LMC performed experiments. JC, EC, PSL, MLT, MBS, JN, SS, IP, MG, EHM, SV, AK, GD, DA, KI, NBH, NC, PL, PP, AGR, SB, RDP, and LAH supervised patient recruitment and sample collection at the respective centers. PBA provided group samples for the Fisher’s exact test and Monte Carlo analysis. LAH, JC, and AGR collected and analyzed clinical data. JC, AGR, SR, and RSG organized the preparation of WES and genome sequencing, and JC and KSA analyzed WES data. KSA analyzed RNA-Seq data and performed genetic analysis. LAH and AMJ conceived the scRNA-Seq experiments, and AMJ analyzed the scRNA-Seq data. KSA analyzed RNA-Seq data, performed the genetic study and designed, processed, and analyzed the Monte Carlo method and Fisher’s exact test data. MB and TAC wrote the manuscript.

## Supplementary Material

Supplemental data

Supplemental data set 1

Supplemental data set 2

Supplemental data set 3

## Version 1 10/25/2022

In-Press Preview

## Version 2 01/03/2023

Electronic publication
