# Supplementary material for: The Notch1/CD22 signaling axis disrupts Treg function in SARS-CoV-2–associated multisystem inflammatory syndrome in children
Source: J Clin Invest. 2023 Jan 3;133(1):e163235. doi: 10.1172/JCI163235 (PMC9797337; doi:10.1172/JCI163235)
Supplement: Supplemental data [file jci-133-163235-s081.pdf]

The Notch1/CD22 signaling axis disrupts Treg cell function in SARS-CoV2-associated multisystem inflammatory syndrome in children

One Sentence Summary: Notch1-CD22 Axis Promotes Immune dysregulation in MIS-C

Mehdi Benamar[1,2], Qian Chen[1,2], Janet Chou[1,2*], Amélie M Julé[1,2,3*], Rafik Boudra[4], Paola Contini[5,6], Elena Crestani[1,2], Peggy S. Lai[7,8], Muyun Wang[1,2], Jason Fong[1,2], Shira Rockwitz[9], Pui Lee[1,2], Tsz Man Fion Chan[1,2], Ekin Zeynep Altun[10], Eda Kepenekli[11], Elif Karakoc-Aydiner[12], Ahmet Ozen[12], Perran Boran[13], Fatih Aygun[14], Pinar Onal[14], Ayse Ayzit Kilinc Sakalli[14], Haluk Cokugras[14], Metin Yusuf Gelmez[15], Fatma Betul Oktelik[15], Esin Aktas Cetin[15], Yuelin Zhong[1,2], Maria Lucia Taylor[1,2], Katherine Irby[16], Natasha B. Halasa[17], Elizabeth H Mack[18] Overcoming COVID-19 Investigators[19], Sara Signa[20], Ignazia Prigione[21], Marco Gattorno[21], Nicola Cotugno[22.23], Donato Amodio[22], Raif S. Geha[1,2], Mary Beth Son[1,2], Jane Newburger[24,2] Pankaj B. Agrawal[25,9,2], Stefano Volpi[20], Paolo Palma[22,23], Ayca Kiykim[26], Adrienne G. Randolph[27,2], Gunnur Deniz[15], Safa Baris[12], Raffaele De Palma[5,28,29], Klaus Schmitz-Abe[1,2,9], Louis-Marie Charbonnier[1,2], Lauren A. Henderson[1,2], Talal A. Chatila[1,2,30]

[1]Division of Immunology, Boston Children's Hospital, Boston, Massachusetts, USA; [2]Department of Pediatrics, Harvard Medical School, Boston, Massachusetts, USA; [3]Department of Biostatistics, Harvard T.H. Chan School of Public Health, Boston, MA,USA [4]Brigham and Women Hospital, department of dermatology, Harvard Medical School, Boston, Massachusetts, USA, [5]Unit of Clinical Immunology and Translational Medicine, IRCCS Ospedale Policlinico San Martino, Genoa, Italy; [6] Department of Cardiology, Boston Children's Hospital, Boston, Massachusetts, USA [7]Division of Pulmonary and Critical Care, Massachusetts General Hospital, Boston, Massachusetts,

USA; [8]Department of Medicine, Harvard Medical School, Boston, Massachusetts, USA; [9] The Manton Center for Orphan Disease Research, Boston Children's Hospital, Boston, USA [10] Ministry of Healthy, Marmara University Education and Training Hospital, Department of Pediatrics, Istanbul, Turkey; [11] Marmara University, Faculty of Medicine, Division of Pediatric Infectious Diseases, Istanbul, Turkey, [10] Marmara University, Faculty of Medicine, [12]Division of Pediatric Allergy and Immunology. The Isil Berat Barlan Center for Translational Medicine, Istanbul, Turkey, [13] Marmara University, Faculty of Medicine, Division of Social Pediatrics, Istanbul, Turkey [14] Division of Pediatric Allergy and Immunology, Faculty of Medicine, Istanbul University-Cerrahpasa, Istanbul, Turkey, [15]Department of Immunology, Aziz Sancar Institute of Experimental Medicine (Aziz Sancar DETAE), Istanbul University, Istanbul, Turkey; [16] Arkansas Children's Hospital, Little Rock, [17] Division of Pediatric Infectious Diseases, Department of Pediatrics, Vanderbilt University Medical Center, [18] Division of Pediatric Critical Care Medicine, Medical University of South Carolina, Charleston.[19]A complete list of the Overcoming COVID-19 Investigators is provided in the Supplementary, [20]DINOGMI, Università degli Studi di Genova, Genova, Italy and Center for Autoinflammatory Diseases and Immunodeficiencies, IRCCS Istituto Giannina Gaslini, Genova, Italy, [21] Center for Autoinflammatory Diseases and Immunodeficiencies, IRCCS Istituto Giannina Gaslini, Genova, Italy, [22] Clinical and Research Unit of Clinical Immunology and Vaccinology, Bambino Gesù Children's Hospital, IRCCS, Rome, Italy,[23] Chair of Pediatrics, Department of Systems Medicine, University of Rome "Tor Vergata", Roma, Italy. [24] Department of Cardiology, Boston Children's Hospital, Boston, Massachusetts, USA, [25]Division of Newborn Medicine and Genetics & Genomics, Department of Pediatrics, Boston

Children's Hospital, Harvard Medical School, Boston, Massachusetts, USA [26] Division of Pediatric Allergy and Immunology, Faculty of Medicine, Istanbul University-Cerrahpasa, Istanbul, Turkey; [27] Department of Anesthesiology, Critical Care, and Pain Medicine, Boston Children's Hospital, Boston, Massachusetts, USA; [28]Department of Internal Medicine (DIMI)-University of Genoa, Genoa; [29]CNR-Institute of Biomolecular Chemistry (IBC), Via Campi Flegrei 34, 80078 Pozzuoli, Napoli, Italy;

[30]Lead Contact

* These authors contributed equally

Corresponding Author: Talal A. Chatila, M.D., M.Sc.**,** Division of Immunology, Boston Children's Hospital**,** Department of Pediatrics, Harvard Medical School, Boston, MA, USA. Email: talal.chatila@childrens.harvard.edu

Experimental Procedures

Single-cell RNA sequencing. Cryopreserved PBMCs were thawed in plain RPMI (HyClone) pre-warmed to 37°C, washed in PBS (HyClone) and resuspended in FACS buffer (PBS with 1.5% FBS (Genesee Scientific) and 2.5 mM EDTA (Invitrogen)) for CD4 T cell enrichment through negative selection (Miltenyi Biotec). Samples were studied in 2 independent experiments: experiment 1 included 3 pediatric controls, 1 pre-treatment MIS-C patient, and 4 post-treatment MIS-C patients; experiment 2 included 1 pediatric control, 2 pre-treatment MIS-C patients, and 1 post-treatment MIS-C patient.

CD4 T cells isolated from each sample were stained with Hashing antibodies targeting CD298 and β2 microglobulin (BioLegend TotalSeq™-C anti-human Hashtag, Clones: LNH-94 and 2M2) for subsequent sample identification. To that end, cells were spun at 500 g for 7 min at 4°C, resuspended into 75 µL Fc block (BioLegend, Cat. No. 422302, 1:20 dilution) and incubated for 10 min at 4°C. 75 µL of hashing antibody (6.7 µg/mL working concentration) were then added and samples were incubated for 30 min at 4°C, with gentle resuspension midway. Cells were washed three times in Hash Staining buffer (BioLegend, Cat. No. 420201) and resuspended in PBS with 0.4% BSA (Sigma, Cat. No. A7030) at a concentration of 1,000 cells per µL. Finally, samples were pooled in equal ratio (7,500 cells per sample) for further processing, thus limiting technical batch effects (1). For the first experiment, the total of 60,000 cells pooled from 8 samples were split across two 10x Genomics chip channels, while the second experiment (30,000 cells from 4 samples) was loaded on one single channel. Cells were encapsulated, barcoded and lysed to enable the generation of cDNA libraries for transcriptome and HTO sequencing

using the 10x Genomics technology (2). Libraries were sequenced on an Illumina NovaSeq 6000.

Single-cell RNA sequencing clustering analyses. Sequencing data from each 10x run were processed with the CellRanger pipeline (10x Genomics) for demultiplexing and gene alignment (2). The resulting raw count matrices were imported in R (v4.0.2 and above) using Seurat (v4.0.3) (3). Data from all 3 runs were merged into one Seurat object. Genes detected in <1 per 10,000 cells were filtered out, leaving a transcriptomic coverage of 21,675 genes. High quality cells with >1400 unique molecular identifiers (UMIs), >700 genes, a log10(gene) to log10(UMI) ratio >0.84 and mitochondrial to nuclear gene ratio <0.08 were retained for downstream analyses. Quality control revealed no significant batch effect: similar distributions were observed for the metrics mentioned above across different runs and experiments.

HTO data were normalized using centered log ratios before applying Seurat::HTODemux() with the clara method and a positive quantile cutoff of 0.98. Doublets and cells with unclear HTO assignment were excluded (Stoeckius et al., 2018). Transcriptomic data for the remaining cells were normalized using Seurat:: SCTransform() and regressing out the effects of the mitochondrial gene ratio, number of UMIs and number of genes detected. Principal components (PCs) were calculated from the top 2000 variable features to reduce the data before mapping to a reference PBMC dataset using Azimuth (3). Cells mapped to CD4 T cell subsets were retained while contaminating lymphocytes were excluded, leaving a total of 29,754 Azimuth-annotated CD4 T cells for downstream analyses. SCT normalization and PCs calculation were repeated at this stage, to account for the top 3000 variable features after exclusion of

TRAV, *TRAJ*, *TRBV* and *TRBJ* genes, thereby enabling cell clustering by transcriptomic profile independently of clonal identity. To control for inter-sample variability, the data were integrated by source sample using Harmony (4). Uniform manifold approximation and projection (UMAP) coordinates were then computed from the first 50 components of the harmony reduction, and graph-based clustering analysis was run on the first 40 components using Seurat::FindNeighbors() and Seurat::FindClusters(). A resolution of 0.6 was retained to define clusters. Seurat-defined clusters were manually annotated, with an initial coarse characterization based on the abundance of cells classified as naïve versus effector/memory by Azimuth. In parallel, genes significantly upregulated in each cluster were identified with Seurat::FindAllMarkers() using the Wilcoxon rank sum test. Significance was defined as a p-value of <0.05 and a log2 fold change (LFC) in gene expression >0.25. Heatmaps were generated with Seurat::DoHeatmap() applied to the scaled SCT data on a random subsample of 100 cells per cluster. To confirm upregulation of NF-κB genes, a signature score for the TNFα signaling via NF-κB geneset, sourced from the MSigDB Hallmark collection (5), was calculated at the single-cell level using Seurat::AddModuleScore().

Pseudobulk differential expression analyses (DEA). For pseudobulk differential expression analyses (DEA), gene expression level data was aggregated at the patient level for each subset of interest, namely Tregs and activated Tconv. For this analysis, we considered as Treg any cell assigned to Seurat Cluster 15 (*FOXP3*-expressing cells) or annotated as Treg by Azimuth, which added up to 1,925 Treg cells across all 12 patients. Similarly, we considered as activated Tconv any cell assigned to Seurat Clusters 9 to 14 and annotated as CD4 TCM, CD4 TEM, CD4 CTL or CD4 Proliferating by Azimuth (6,674

cells). PC analyses of the aggregated transcriptomic data highlighted healthy control 4 as a strong outlier among both Treg and activated Tconv subsets, leading us to exclude this patient from pseudobulk DEA. Independent pairwise analyses contrasting each of the 3 patient groups (MIS-C pre-treatment, MIS-C post-treatment and control) were run using DESeq2 (version 1.34.0). Log2 fold change (LFC) values were corrected using the apeglm shrinkage estimator and used as input for gene set enrichment analyses (GSEA) against the MSigDB Hallmark collection, performed with clusterProfiler (version 4.2.2). Heatmaps of gene expression for significant genes (defined as an adjusted p-value <0.05) were generated from the centered rlog-normalized count data using pheatmap (version 1.0.12).

Gene pathway analysis using the Fischer and Monte-Carlo tests. To identify if a pathway is relevant to MIS-C or acute-COVID-19 (mild and severe pediatrics patients), a comparison between MIS-C or acute-COV19 and the eight databanks described above was performed using the following steps below. To minimize false positive and artifactual results, all samples were processed using the same pipeline, Variant Explorer (VExP) (6), starting with their raw data (Fastq files).

Step 1 (fastq to vcf file): Raw data were processed to obtain vcf files using the human reference assembly 19, BWA (alignment, v0.7.17), PICARD (mark/delete duplicates, v2.23.3), SAMTOOLS (variant calling, v1.10), and GATK (multi-sample variant calling, v4.1.8.1). When only bam files were available, PICARD (v2.23.3) was used to revert to fastq files. Further, ANNOVAR (2020Apr) and custom VExP scripts were used to add annotations from relevant genetic databases into each vcf file.

Step 2 (Variant filtering): Variant analysis was performed in each family based on three filtering criteria: first, include variants predicted by ANNOVAR to have a potential functional coding consequence, including stop gain or loss, splice site disruption, indel, and nonsynonymous. Second, variants are filtered based on allele frequency in control populations (gnomAD, ExAC, EVS, 1000GP, and internal data from 8114 unaffected individuals from BCH). Heterozygous/hemizygous variants were included if minor allele frequency (MAF) was <0.0005 (0.05%) in any database. In comparison, homozygous variants were included only if MAF was ≤0.00005 (0.005%) and for compound heterozygous models the MAF cutoff was ≤0.01 (1%) with no homozygous variant reported in any database. The variants were further prioritized to include those with read depth ≥10X, alternative depth ≥5X, allele balance ≥0.20, and deleterious prediction (4 or more of 23 softwares, including PolyPhen, SIFT, FATHMM, and CADD).

Step 3 (gene-enrichment): A gene-enrichment test was performed to identified rare pathogenic variants and lost/gain of function (stop-lost/gain, frameshift deletions/insertions and canonical splicing sites) using 8,626 pathways from Gene-Ontology [Gene-Ontology database: http://geneontology.org/] and KEGG [KEGG database: https://www.genome.jp/kegg/] databases (8,299 and 327 respectively). The Frequencies (families) of these rare coding pathogenic variant gene were calculated for each pathway using 3 different genetic models: a) Homozygous variants, b) Heterozygous variants and c) Homozygous and/or 2 or more heterozygous variants in the same gene with a minimum distance between them of 100 base pairs (compound heterozygous filters). P values were calculated using 2 methods: traditional Fisher test

(two sided) and Monte-Carlo method. The expectation for one event (pathway) using Monte Carlo method is described by the following formula:

$$F^k = 1/N \sum_{i=1}^{N=10,000} X_i^k$$

Where "F" represents the number of families with rare pathogenic variants in the "k" pathway (k=1:8,626 pathways) and "X" is a random control group with the same number of samples of the comparison group, for MIS-C, 39 samples and for acute-19, 24 samples. Independent samples were taken random using a uniform distribution and 4682 samples described **above.** "N" is the total number of independent simulations (10,000 in total). The use of independent samples was very important to establish fairness in our tests, so then we use only one sample per family (probands).

Transcriptome Profiling. Treg cells were isolated from either *Foxp3*^{EGFPcre} or Foxp3^{EGFPCre} *Rosa26*$^{N1c/+}$ mice by cell sorting. mRNA was isolated using Qiagen RNeasy mini kit (Qiagen). RNA was then converted into double-stranded DNA (dsDNA), using SMART-Seq v4 Ultra Low Input RNA kit (Clontech). dsDNA was then fragmented to 200-300 bp size, using M220 Focused-ultrasonicator (Covaris), and utilized for construction of libraries for Illumina sequencing using KAPA Hyper Prep Kit (Kapa Biosystems). Libraries were then quantified using Qubit dsDNA HS (High Sensitivity) Assay Kit on Agilent High Sensitivity DNA Bioanalyzer.

Gene-level read counts were quantified using feature Counts and the latest Ensembl mouse annotation (GRCm38.R101). Raw data were trimmed using Trimmomatic (version 0.39, default parameters), tool for Illumina NGS data. To identify differentially expressed genes, we used 3 algorithms: DESeq2 (version 1.26.0), edgeR (version 3.28.1) and Lima

(3.42.2) Bioconductor packages with default parameters. Count tables were normalized to TPM (Transcripts per Million) for visualizations and QC. Sample clustering, path analyses and integration of the results were performed using a custom-made pipeline available upon request (Variant Explorer RNAseq). Transcripts were called as differentially expressed when the adjusted p values were below 0.05, fold-changes over ±1.5 and false discovery rate (FDR) were below 0.05. For our path analyses, we tested 10,715 biological pathways from KEGG and GO annotations. We filtered the results using an adjusted p value below 0.001.

Supplementary Figure Legends

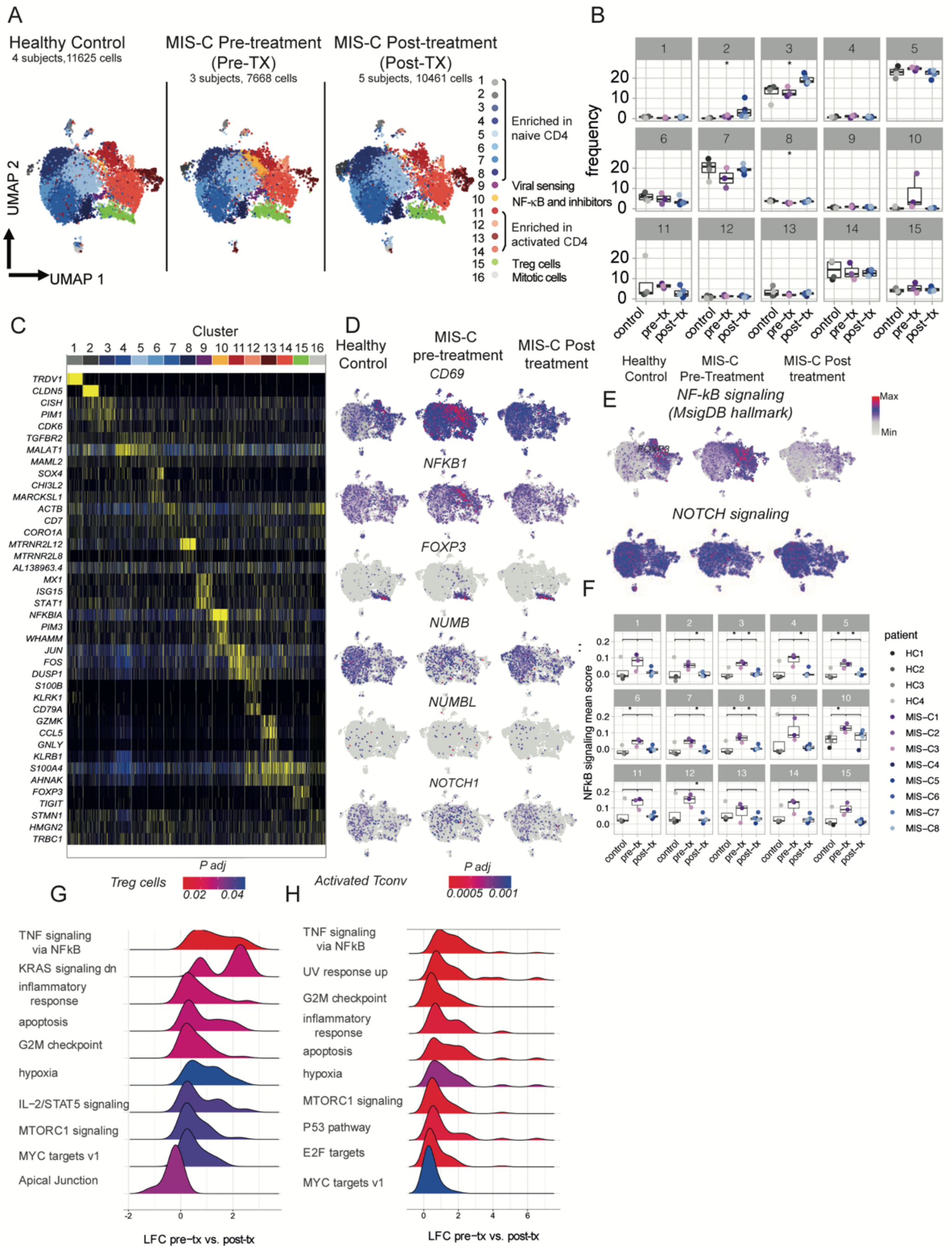

Fig. S1. Single-cell transcriptomic analyses of circulating CD4$^+$ T cells from control, pre- and post-treatment MIS-C subjects. A. Uniform manifold approximation and projection (UMAP) of normalized and harmonized dataset, split by disease group and color-coded by cluster. Clusters were delineated using Seurat. **B**. Frequencies (%) of

each cluster among total CD4^{+} T cells for each patient. **C**. Heatmap showing expression of the top genes in each cluster, as determined using Seurat. **D**. UMAP split by disease group and color-coded by expression of *CD69*, *NFKB1*, *FOXP3, NUMB, NUMBL and NOTCH1* at the single-cell level. **E.** UMAP split by disease group and color-coded by single-cell score for the TNFα signaling via NF-κB gene set (MSigDB Hallmark) and NOTCH signaling. **F**. TNFα signaling via NF-κB gene set mean score, averaged per cluster and patient. Multiple T-test comparisons significant at an FDR of 0.05 are indicated with a star. **G**, **H.** LFC distributions of genes belonging to each of the corresponding enriched hallmarks. Gene set enrichment analysis (GSEA) was run against the MSigDB hallmark database using ranked LFC derived from pseudobulk DEA of pre-treatment MIS-C versus control subjects in Tconv .

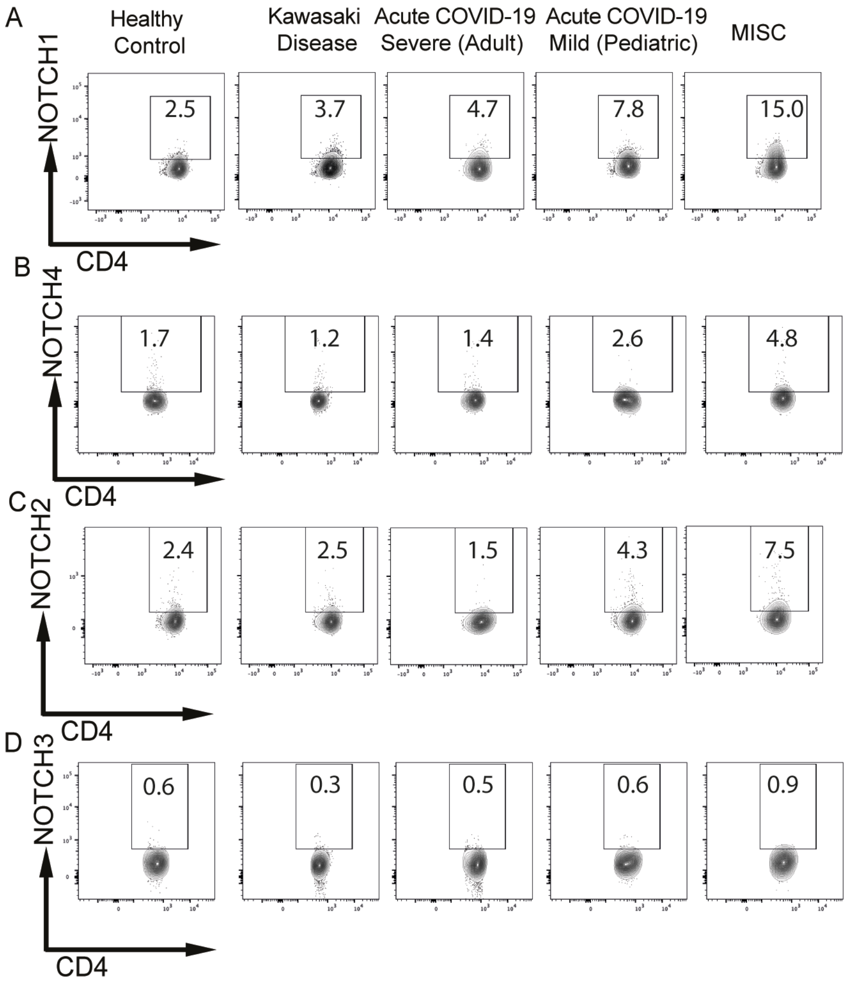

Figure S2: Flow cytometry analysis of Notches receptors expression on MISC Tconv: A-D. Flow cytometric analysis of Notch1 **(A)**, Notch4 **(B)**, Notch2 **(C)** and Notch3 **(D)** expression in $CD4^+$ Tconv cells of healthy control subjects, and patients with Kawasaki disease, adult subjects with severe COVID-19, pediatric subjects with mild or severe COVID-19 and MIS-C subjects.

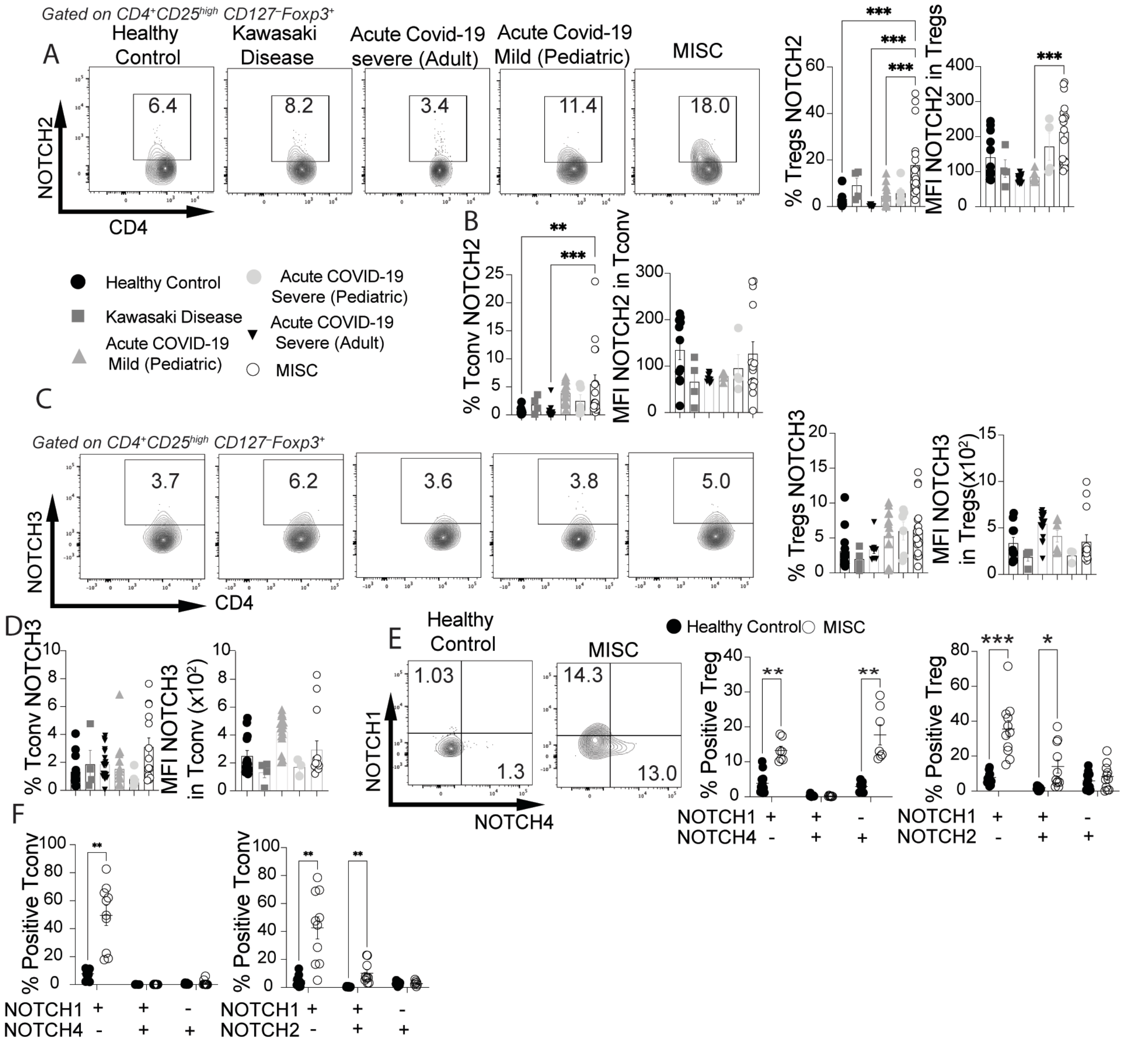

Fig. S3. **Notch receptor expression on circulating CD4⁺ Treg and Tconv cells in MIS-C**. **A to D.** Flow cytometric analysis, cell frequencies and mean fluorescence intensity (MFI) of Notch2 (**A,B**) and Notch3 expression (**C,D**) in CD4⁺ Treg and Tconv cells of healthy control subjects, and patients with Kawasaki disease, adult subjects with severe COVID-19, pediatric subjects with mild or severe COVID-19 and MIS-C subjects. **E-F.** Flow cytometric analysis, and cell frequencies of co-expression of Notch1 and Notch2 and Notch1 and Notch4 on circulating Treg cells (E) and Tconv (F) of MIS-C subjects**.**

Each symbol represents one subject. Numbers in flow plots indicate percentages. Error bars indicate SEM. Statistical tests: *P<0.05, **P<0.01, ***P<0.001, ****P<0.0001 by one-way ANOVA with Dunnett's post hoc analysis (**A to F**).

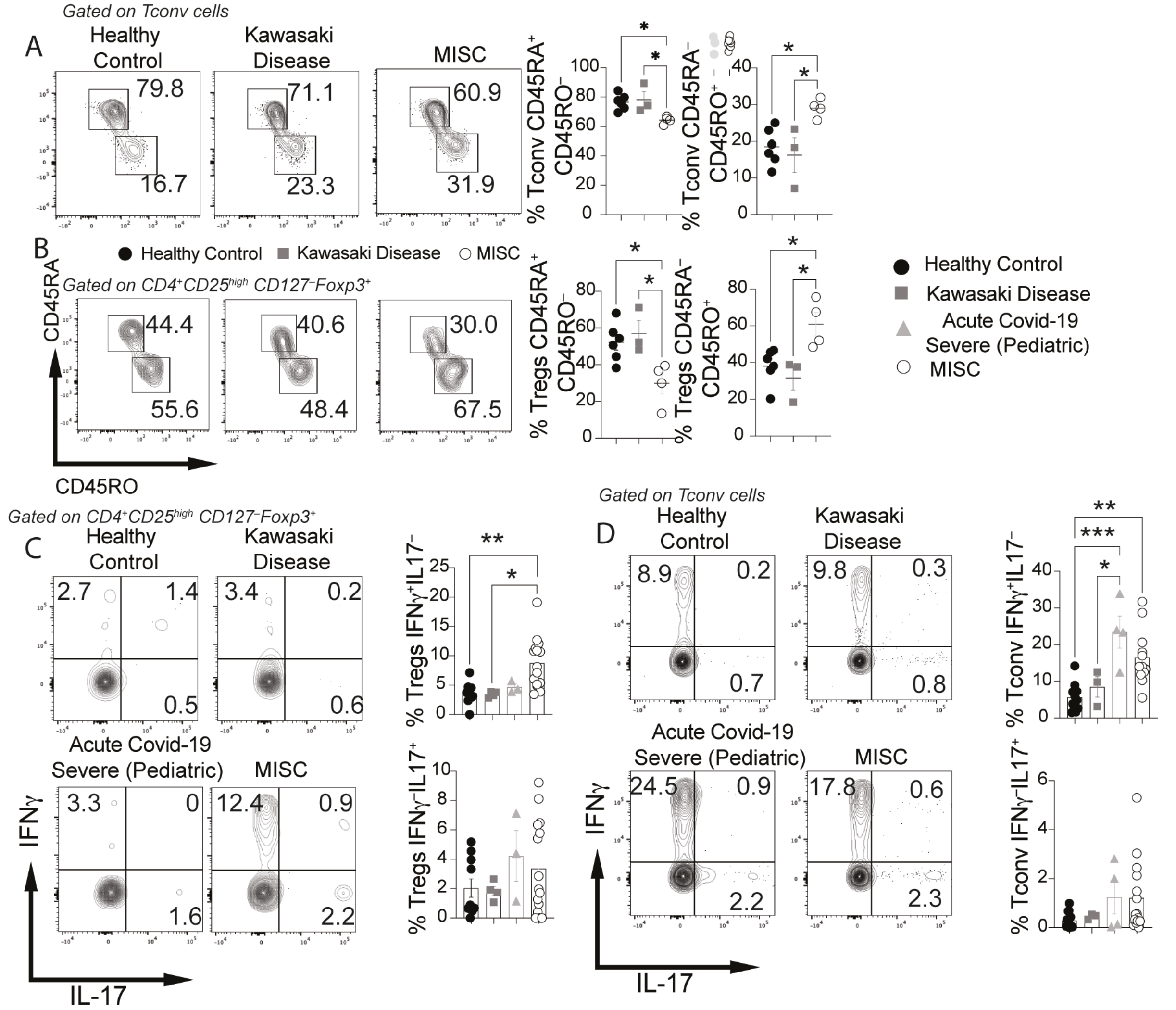

Fig. S4. **Characterization of circulating CD4⁺ Treg and Tconv cells in MIS-C**. **A,B**. Flow cytometric analysis and cell frequencies of T cell activation state markers (CD45RA, CD45RO) on Tconv (**A**) and Treg (**B**) cells of the respective subject groups. **C and D.** Flow cytometric analysis and frequencies of IFNγ and IL-17-expressing Treg (**C**) and Tconv (**D**) cells of the respective subject groups. Each symbol represents one subject. Numbers in flow plots indicate percentages. Error bars indicate SEM. Statistical tests: *P<0.05, **P<0.01, ***P<0.001, ****P<0.0001 by one-way ANOVA with Dunnett's post hoc analysis (**A to D**).

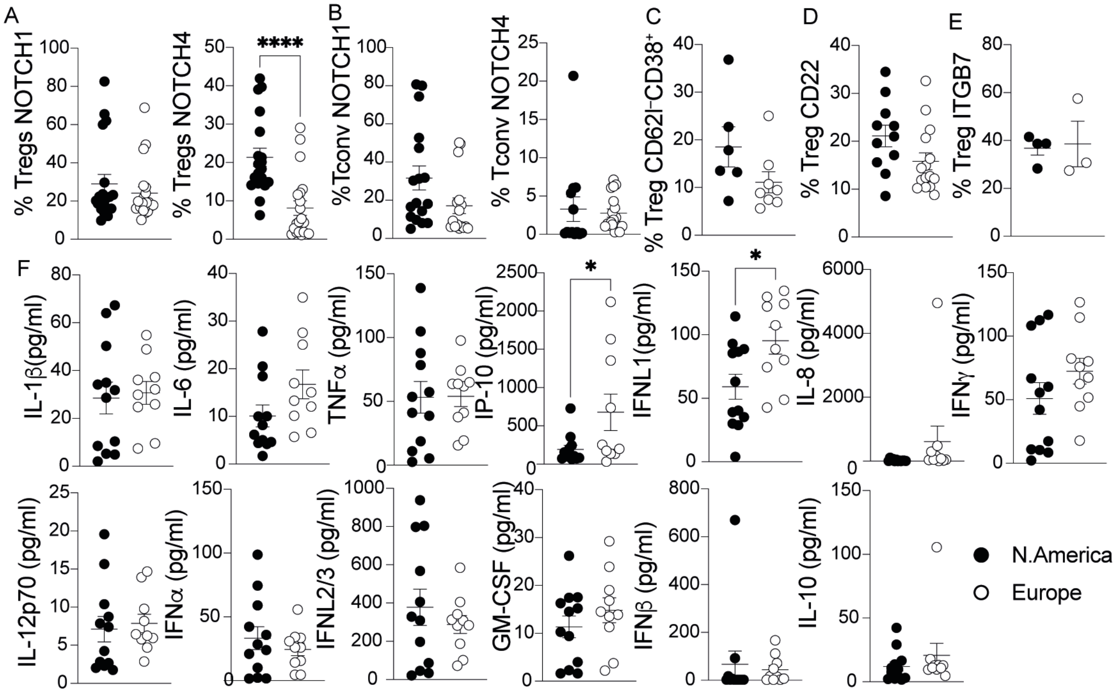

Figure S5: Comparison of MISC patients' characteristics based on the country of residency: **A**-**B**. Cell frequencies of Notch1 and Notch4 expression in CD4$^+$ Treg (**A**) and Tconv cells (**B**) of MISC from North America or Europe. **C,D,E**. Cell frequencies of mucosally imprinted (CD62l$^-$CD38$^+$) (**C**), CD22 (**D**) and ITGB7 (**E**) on Treg cells form the respective groups. **F**. serum concentrations of IL-1β, IL-6, TNF, IP-10, IFNL1, IL-8, IL12p70, IFNα, IFNλ2/3, IFNγ and IL-10, in the respective patient group subjects. Each symbol represents one subject. Error bars indicate SEM. Statistical tests: *P<0.05, ****P<0.0001 by student-t-test (**A and F**).

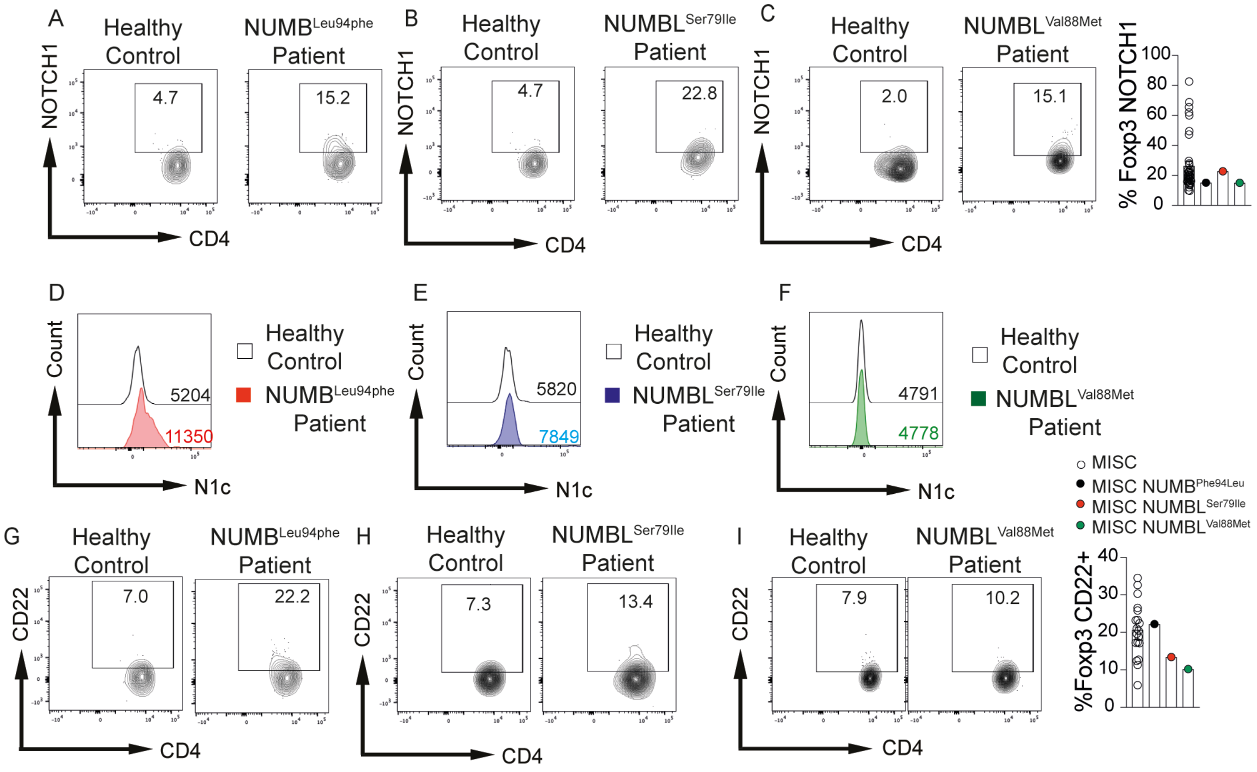

Fig. S6: Characterization of circulating CD4⁺ Treg in MIS-C with specific mutation in NUMB/NUMBL. A to I. Flow cytometric analysis and cell frequencies of Notch1 (**A to C**), N1c expression (**D to F**) and CD22 (**G to I**) in CD4⁺ Treg of healthy control subjects, and MIS-C patients with a specific mutation in $NUMB^{leu94phe}$ (**A,D,G**), $NUMBL^{Ser79Ile}$ (**B,E,H**) and $NUMBL^{Val88Met}$ (**C, F, I**).

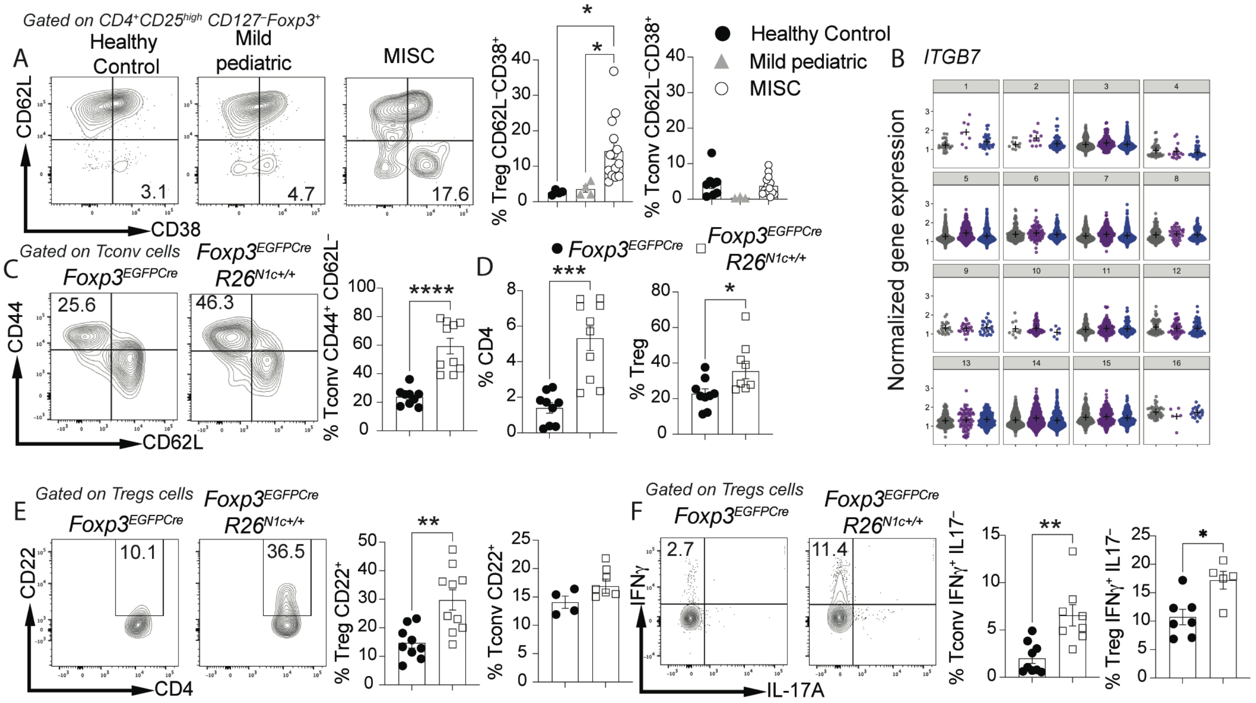

Fig. S7: Attributes of mucosal T cells in MIS-C and Poly I:C-treated *Foxp3*^EGFPCre^*R26*^N1c/+^ mice. A. Flow cytometric analysis and graphical representation of mucosal imprinted (CD62L⁻CD38⁺) Treg and Tconv cells in healthy control subjects, pediatric subjects with mild Covid-19 and MIS-C patients. **B**. Relative *ITGB7* gene expression in cell clusters of healthy control subjects (gray) and in MIS-C patients pre (purple) and post-treatment (blue) inferred from scRNA-seq analysis. **C and D**. Flow cytometric analysis and graphical representation of colonic T cell ($CD3^+CD4^+$), Treg ($CD3^+CD4^+Foxp3^+$) and activated Tconv ($CD4^+CD44^+CD62L^-$) cells of $Foxp3^{EGFPCre}$ and $Foxp3^{EGFPCre}R26^{N1c/+}$ mice subjected to Poly I:C treatment. **E**. Flow cytometric analysis and graphical representation of colonic $CD22^+$ Treg and Tconv cells of $Foxp3^{EGFPCre}$ and $Foxp3^{EGFPCre}R26^{N1c/+}$ mice subjected to Poly I:C treatment. **F**. Flow cytometric analysis and graphical representation of IFNγ and IL-17 expressing Tconv and Treg cells of $Foxp3^{EGFPCre}$ and $Foxp3^{EGFPCre}R26^{N1c/+}$ mice subjected to Poly I:C treatment. Each

symbol represents one human subject (**A**), one cell (**B**) or one mouse (**C to F**). Numbers in flow plots indicate percentages. Error bars indicate SEM. Statistical tests: One-way ANOVA with Dunnett's post hoc analysis (**A,C to F**) Two-way ANOVA with Sidak's post hoc analysis (**B**)**;**. *P<0.05, **P<0.01, ***P<0.001, ****P<0.0001.

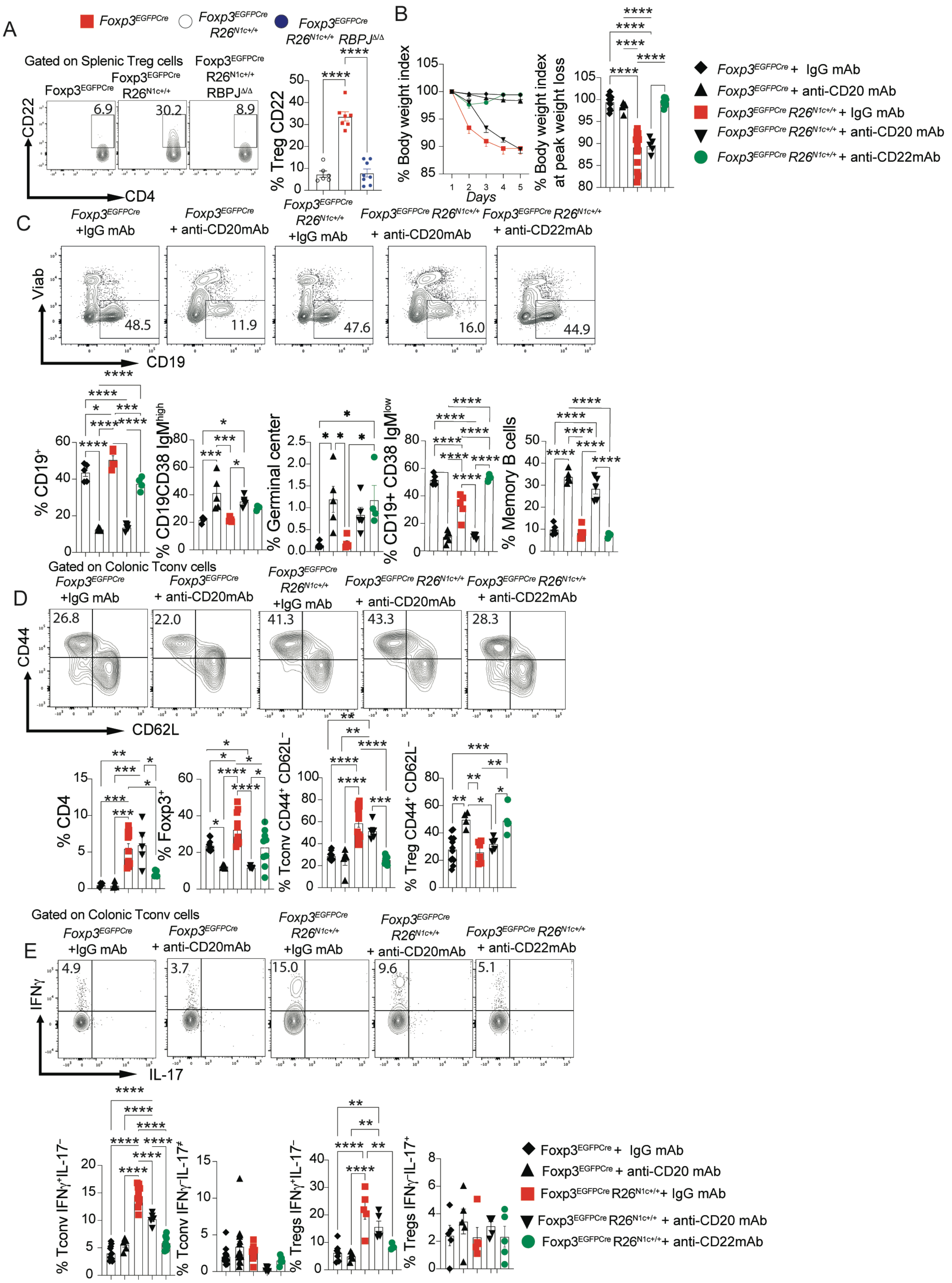
A
Foxp3EGFPCre
Foxp3EGFPCre R26N1c+/+
Foxp3EGFPCre R26N1c+/+ RBPJΔ/Δ
Gated on Splenic Treg cells
CD22
CD4
% Treg CD22
B
% Body weight index
Days
% Body weight index at peak weight loss
Foxp3EGFPCre + IgG mAb
Foxp3EGFPCre + anti-CD20 mAb
Foxp3EGFPCre R26N1c+/+ + IgG mAb
Foxp3EGFPCre R26N1c+/+ + anti-CD20 mAb
Foxp3EGFPCre R26N1c+/+ + anti-CD22mAb
C
Viab
CD19
% CD19+
% CD19CD38 IgMhigh
% Germinal center
% CD19+ CD38 IgMlow
% Memory B cells
Gated on Colonic Tconv cells
D
CD44
CD62L
% CD4
% Foxp3+
% Tconv CD44+ CD62L−
% Treg CD44+ CD62L−
Gated on Colonic Tconv cells
E
IFNγ
IL-17
% Tconv IFNγ+IL-17−
% Tconv IFNγ−IL-17+
% Tregs IFNγ+IL-17−
% Tregs IFNγ−IL-17+

Fig. S8. Anti-CD22 mAb therapy of Poly I:C-induced disease in *Foxp3*^{EGFPCre}*R26*$^{N1c/+}$ mice is B cell-independent. A. Flow cytometric analysis and frequencies of CD22 expression in Treg cells of *Foxp3*^{EGFPCre}, *Foxp3*^{EGFPCre}*Notch1c*$^{+/-}$ and *Foxp3*^{EGFPCre}*Notch1c*$^{+/-}$*RBPJ*$^{\Delta/\Delta}$ mice. **B.** Weight indices of Poly I:C treated *Foxp3*^{EGFPCre} and *Foxp3*^{EGFPCre}*R26*$^{N1c/+}$ mice co-injected with anti-CD22 mAb or with an anti-CD20mAb. **C**. Flow cytometric analysis and graphical representation of different splenic B cell populations [CD19$^+$, CD19$^+$CD38$^+$IgM^{high}, germinal B cells (GL7$^+$), CD19$^+$CD38+IgM^{low} and memory B cells (IgD$^-$CD27$^+$)] of *Foxp3*^{EGFPCre} and *Foxp3*^{EGFPCre}*R26*$^{N1c/+}$ mice subjected to Poly I:C treatment. **D**. Flow cytometric analysis and graphical representation of colonic T cell (CD3$^+$CD4$^+$), Treg cells (CD3$^+$CD4$^+$Foxp3$^+$) and activated Tconv (CD4$^+$CD44$^+$CD62L$^-$) of Poly I:C treated *Foxp3*^{EGFPCre} and *Foxp3*^{EGFPCre}*R26*$^{N1c/+}$ mice co-injected with anti-CD22 mAb or with an anti-CD20mAb. **E**. Flow cytometric analysis and graphical representation of IFNγ and IL-17 expressing colonic Tconv and Treg cells of Poly I:C treated *Foxp3*^{EGFPCre} and *Foxp3*^{EGFPCre}*R26*$^{N1c/+}$ mice co-injected with anti-CD22 mAb or with an anti-CD20mAb. Numbers in flow plots indicate percentages. Error bars indicate SEM. Statistical tests: One-way ANOVA with Dunnett's post hoc analysis (**A, C to E**), Two-way ANOVA with Sidak's post hoc analysis (**B**); *P<0.05, **P<0.01, ***P<0.001, ****P<0.0001.

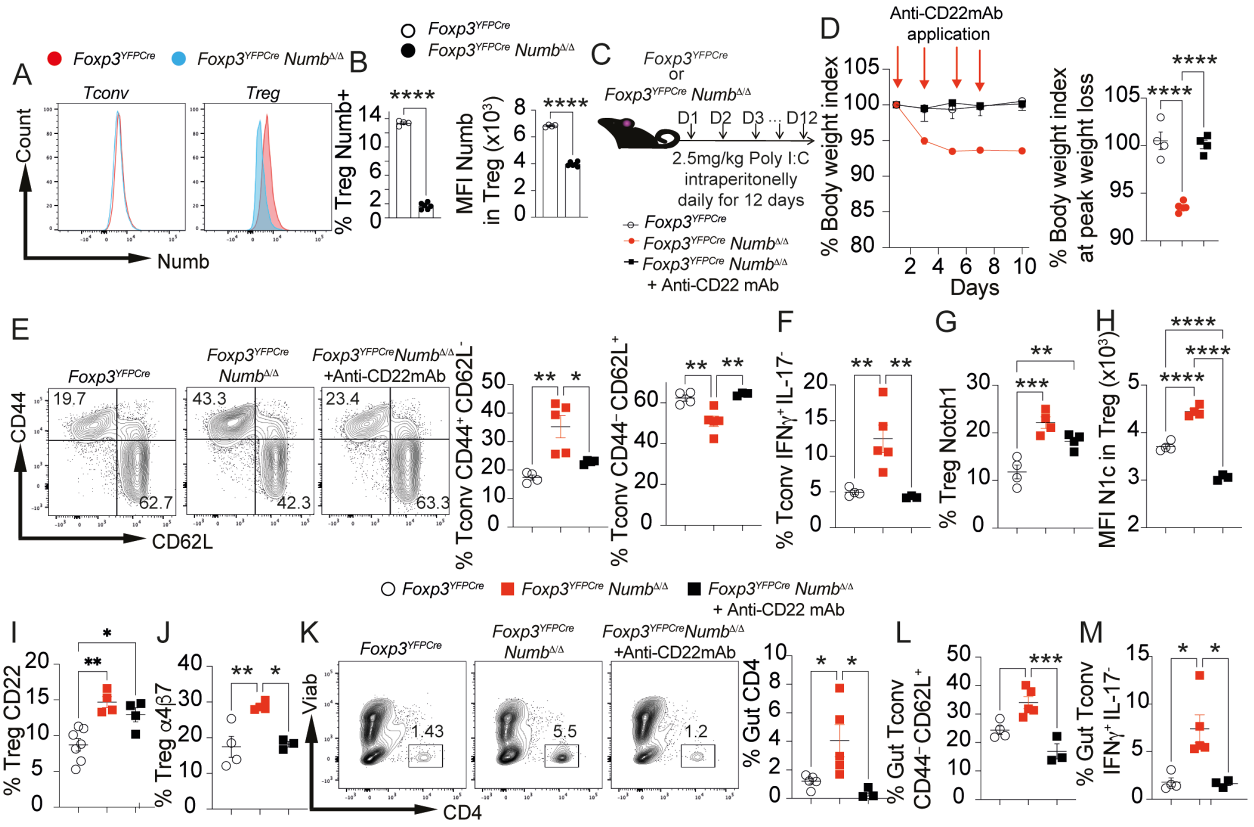

Fig. S9: Poly I:C-induced multiorgan inflammatory disease in ***Foxp3*^{YFPCre}*NUMB*$^{\Delta/\Delta}$ mice. A.** Flow cytometric analysis of Numb expression in Tconv and Treg cells from *Foxp3*^{YFPCre} and *Foxp3*^{YFPCre}*Numb*$^{\Delta/\Delta}$ mice. **B.** Cell frequencies and MFI of Numb expression on Treg cells from the respective mice. **C**. Scheme of mouse Poly IC treatment. **D**. Body weight index change and peak weight loss of the *Foxp3*^{YFPCre} and *Foxp3*^{YFPCre}*Numb*$^{\Delta/\Delta}$ mice treated with Poly IC alone or in combination with anti-CD22 mAb. **E and F**. Flow cytometric analysis and cell frequencies of CD44^{+}CD62L^{-} (**E**) and IFNγ^{+}IL-17^{-} Tconv cells (**F**). **G to J**. frequencies of Notch1^{+} (**G**), MFI of Notch1c^{+} (**H**), CD22 (**I**) and α4β7 (**J**) in Treg cells after Poly I:C treatment alone or in combination with anti-CD22 mAb. **K to M** Flow cytometric analysis and cell frequencies of Gut CD4 (**K**), of CD44^{+}CD62L^{-} (**L**) and IFNγ^{+}IL-17^{-} (**M**) gut Tconv cells. Numbers in flow plots indicate

percentages. Error bars indicate SEM. Statistical tests: One-way ANOVA with Dunnett’s post hoc analysis (**B to H, J to M**); *$P<0.05$, **$P<0.01$, ***$P<0.001$, ****$P<0.0001$.

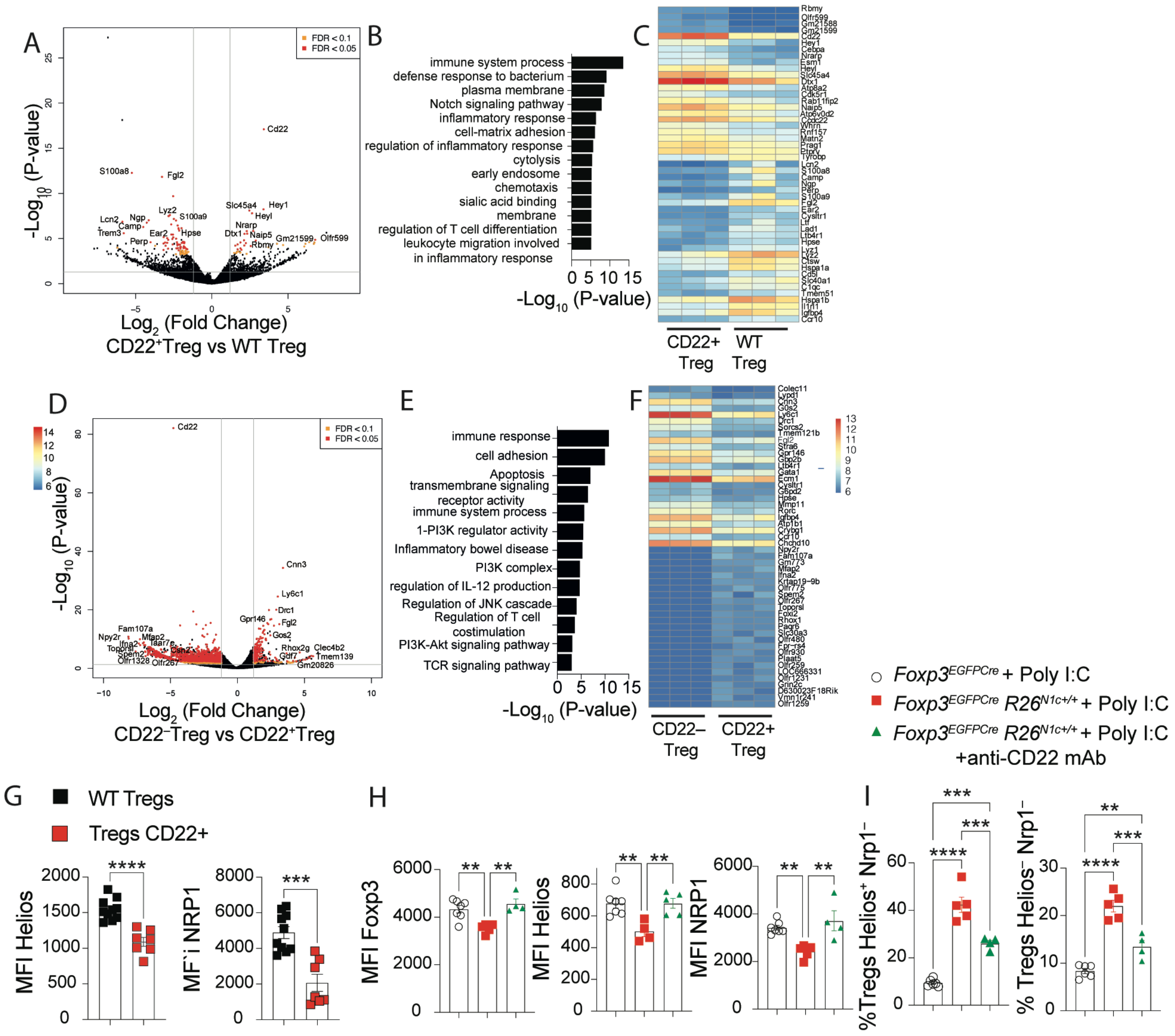

Fig. S10. Treg cell instability in Poly I:C-treated *Foxp3*^{EGFPCre}*R26*$^{N1c/+}$ mice. A to C, Volcano plot (A), pathway analysis (B) and heat map (C) of gene transcripts of WT or CD22+ Treg cells isolated at steady-state from *Foxp3*^{YFPCre} and *Foxp3*^{EGFPCre}*R26*$^{N1c/+}$mice (n=3). **D to F**, Volcano plot (D), pathway analysis (E) and heat map (F) of gene transcripts of CD22$^{–}$ or CD22^{+} Treg cells isolated at steady state from *Foxp3*^{EGFPCre}*R26*$^{N1c/+}$mice. **G,** MFI of splenic Treg cell markers of WT or CD22^{+} Treg cells isolated at steady state from *Foxp3*^{YFPCre} and *Foxp3*^{EGFPCre}*R26*$^{N1c/+}$mice. **H,** Flow cytometric analysis and MFI of splenic Treg cell markers of Poly I:C-treated *Foxp3*^{EGFPCre} and *Foxp3*^{EGFPCre}*R26*$^{N1c/+}$ mice

co-treated with isotype control mAb or anti-CD22 mAb, as indicated. **I**, Cell frequencies of Helios and NRP1 expression on splenic Treg cells in the groups shown in (H). Each symbol represents one mouse. Error bars indicate SEM. Statistical tests: Student-t-test (G), One-way ANOVA with Dunnett's post hoc analysis (H,**I**);**;** **P<0.01, ***P<0.001, ****P<0.0001.

Table S1: Clinical characteristics of patient and control subjects.

	MIS-C<br>N=45	Pedi COVID-19<br>N=50	KD<br>N=5	Pedi Controls<br>N=18
**Patient Demographics**				
Age-yrs (median, IQR)	8.0, 5.0-12.4	13.0, 3.0-16.0	7.0, 1.5-10.5	3.5, 1.0-5.8
Sex (#, % female)	30, 68%	24, 48%	2, 40%	6, 33%
Race & Ethnicity (#, %)				
White, non-Hispanic	25, 56%	43, 86%	3, 60%	15, 83%
Black, non-Hispanic	4, 9%	0, 0%	0, 0%	0, 0%
Hispanic	9, 20%	4, 8%	1, 20%	2, 11%
Asian	0, 0%	0, 0%	1, 20%	1, 6%
Other	2, 4%	1, 2%	0, 0%	0, 0%
Unknown	5, 11%	2, 4%	0, 0%	0, 0%
**Pre-existing Conditions (#, %)**	15, 33%	7, 14%	2, 40%	0, 0%
**BMI kg/m^2 (median, IQR)**	19.3, 16.1-25.6	18.6, 16.9-23.8	17.0, 14.6-20.5	-
**Case Definitions/Criteria (#, %)**				
WHO MIS-C	42, 93%	-	-	-
CDC MIS-C	45, 100%	-	-	-
Complete KD Criteria	6, 13%	-	3, 60%	-
Incomplete KD Criteria	6, 13%	-	2, 40%	-
**Clinical Features (#, %)**				
Fever	45, 100%	36, 72%	5, 100%	-
Rash	22, 49%	1, 2%	5, 100%	-

Conjunctivitis	26, 58%	1, 2%	5, 100%	-
Mucositis	8, 18%	1, 2%	3, 60%	-
Extremity Changes	12, 27%	1, 2%	4, 80%	-
Lymphadenopathy	11, 24%	0, 0%	2, 40%	-
Shock	14, 31%	0, 0%	0, 0%	-
GI Symptoms	43, 96%	10, 20%	2, 40%	-
Abdominal Pain	28, 62%	4, 8%	1, 20%	-
Vomiting	30, 67%	5, 10%	2, 40%	-
Diarrhea	24, 53%	3, 6%	0, 0%	-
Respiratory Symptoms	18, 40%	27, 54%	0, 0%	-
Cough	4, 9%	19, 38%	0, 0%	-
Dyspnea	3, 7%	4, 8%	0, 0%	-
Hypoxia	9,20%	7, 14%	0, 0%	-
Infiltrate on Chest Imaging	5,11%	9, 18%	0, 0%	-
Neurologic Symptoms	6,13%	6, 12%	0, 0%	-
Headache	3,67%	3, 6%	0, 0%	-
Anosmia/Dysgeusia	0, 0%	2, 4%	0, 0%	-
Altered Mental Status	1, 2%	0, 0%	0, 0%	-
CN Palsy	0, 0%	0, 0%	0, 0%	-
Meningismus	1, 2%	0, 0%	0, 0%	-
Seizure	0, 0%	1, 2%	0, 0%	-
**SARS-CoV-2 Testing (#, %)***				
PCR positive	16, 36%	50, 100%	0, 0%	-
Seropositive	41, 91%	6, 12%	0, 0%	-

**Laboratory Features** (median, IQR)				
Lowest ALC x10^3/mL	0.91, 0.53-1.35	1.8, 1.22-3.35	0.87, 0.65-2.57	-
Lowest Hgb g/dL	10.0, 9.2-10.7	12.6, 10.9-13.8	11.0, 8.2-12.1	-
Lowest Plt x10^3/mL	192, 126-236	269, 207-353	340, 156-414	-
Highest CRP mg/dL	16.0, 7.8-24.0	0.3, 0.1-6.3	5.8, 4.1-21.3	-
Highest Ferritin ng/mL	502, 305-1134	143, 70-326	175, 134-779	-
Highest D-dimer mcg/mL	3.1, 1.5-6.2	0.8, 0.5-1.4	1.7, 1.2-3.9	-
Highest ALT U/L	40, 22-79	31, 17-48	50, 41-133	-
Highest Cr mg/dL	0.47, 0.39-0.61	0.54, 0.26-0.68	0.34, 0.27-0.60	-
Highest BNP pg/mL	1118, 185-2150	34, 13-130	35, 13-422	-
Highest Troponin ng/mL	0.02, 0.01-0.11	0.01, 0.01-0.02	0.01, 0.01-0.01	-
**Cardiovascular Features (#, %)**▲				
EF <55%	16, 36%	-	0, 0%	-
Coronary Artery Dilation	3, 7%	-	0, 0%	-
Coronary Artery Aneurysm	3, 7%	-	2, 40%	-
**Clinical Interventions (#, %)**				
ICU Admission	14, 31%	2, 4%	0, 0%	-
Vasopressors	9, 20%	0, 0%	0, 0%	-
Supplemental O2	8, 18%	8, 16%	0, 0%	-
CPAP	0, 0%	3, 6%	0, 0%	-
BiPAP	5, 11%	1, 2%	0, 0%	-
Mechanical Ventilation	1, 2%	0, 0%	0, 0%	-

**Immunomodulatory Treatments (#, %)**▼				
Any Immunomodulatory Treatment	41, 91%	3, 6%	4, 80%	-
IVIG	37, 82%	1, 2%	4, 80%	-
Glucocorticoids	31, 69%	2, 4%	2, 40%	-
Anakinra	5, 11%	0, 0%	1, 20%	-
Other	3, 7%	0, 0%	0, 0%	-

MIS-C, multisystem inflammatory syndrome in children; pedi, pediatric; COVID-19, coronavirus disease 2019; KD, Kawasaki disease; yrs, years; WHO, World Health Organization; CDC, Centers for Disease Control and Prevention; GI, gastrointestinal; CN, cranial nerve; PCR, polymerase chain reaction; ALC, absolute lymphocyte count; Hgb, hemoglobin; Plt, platelet count; CRP, C-reactive protein; ALT, alanine aminotransferase; Cr, creatinine; BNP, B-type natriuretic peptide; EF, ejection fraction; ICU, intensive care unit; O2, oxygen; CPAP, continuous positive airway pressure; bilevel positive airway pressure; IVIG, intravenous immunoglobulin

*42/45 MIS-C patients were PCR tested for SARS-CoV-2. 44/45 MIS-C patients underwent serologic testing for SARS-CoV-2. All children with acute COVID-19 had PCR testing and 8 had serologic testing. All KD patients had PCR and serologic testing.

▲All children with MIS-C and KD had an echocardiogram preformed.

▼Immunomodulatory treatment before biosample collection. Pre-treatment samples were obtained from 5/33 MIS-C and 1/5 KD patients.

Table S2: Antibodies used for flow cytometry

Mouse antibodies			
Markers	company	catalogue	Clone
Foxp3	Thermofisher	48-5773-82	FJK-16S
IFNγ	Biolegend	505825	XMG1.2
Helios	Thermofisher	47-9883-42	22F6
CD4	Biolegend	100451	GK1.5
CD3	Biolegend	100203	17A2
IL-17	Biolegend	506922	TC11-18H10.1
CD45	Biolegend	103140	30-F11
Notch4	Biolegend	128407	HMN4-14
CD279	Thermofisher	12-9985-82	J43
CD44	Biolegend	103032	IM7
CD62L	Biolegend	104412	MEL-14
N1c	Biolegend	629106	mN1A
$\alpha 4\beta 7$	Biolegend	120606	DATK32
p-Erk	Biolegend	369506	6B8B69
p-PLCγ	Biolegend	612404	A17025A
pS6	CST	5316	D57.2.2E
p-Akt^{s473}	BD	560378	M89-61
p-Akt^{T308}	BD	558375	J1-223.371
CD22	Biolegend	126112	OX-97
Human antibodies			
CD3	Biolegend	300318	HIT3a,

CD4	Biolegend	300530	RPA-T4
Foxp3	Thermofisher	48-4776-42,56-4716-41	PCH-101
Notch1	BD Pharmingen	566023	HMN1-519
Notch2	BD Pharmingen	742291	HMN2-25
Notch3	BD Pharmingen	744828	HMN3-21
Notch4	BD Pharmingen	563269	HMN4-2
CD25	Thermofisher	12-0259-42	BC96
CD127	Biolegend	351320	A019D5
IFNγ	BD Biosciences	560741	4S.B3
ITGB7	BD	551082	FIB504
CCR7	Biolegend	353208	G043H7
CD38	Biolegend	397114	S17015A
CD22	Biolegend	302516	HIB22
CD45RA	Biolegend	304134	HI100
CD45RO	Biolegend	304236	UCHL1
IL17	Biolegend	512315	BL168
CD62L	Biolegend	304810	DREG-56
Purified anti-CD22	Biolegend	302502	HIB22

Overcoming COVID-19 Investigators

(Listed in PubMed, and ordered by U.S. State)

The following study group members were all closely involved with the design, implementation, and oversight of the Overcoming COVID-19 study.

Alabama: Children's of Alabama, Birmingham. Michele Kong, MD.

Arizona: University of Arizona, Tucson. Mary Glas Gaspers, MD; Katri V. Typpo, MD.

Arkansas: Arkansas Children's Hospital, Little Rock. Ronald C. Sanders Jr., MD, MS; Katherine Irby, MD.

California: Children's Hospital of Orange County, Orange County. Adam J. Schwarz, MD.

California: Miller Children's & Women's Hospital Long Beach, Long Beach. Christopher J. Babbitt, MD.

California: UCSF Benioff Children's Hospital Oakland, Oakland. Natalie Z. Cvijanovich, MD.

California: UCSF Benioff Children's Hospital, San Francisco. Matt S. Zinter, MD

Colorado: Children's Hospital Colorado, Aurora. Aline B. Maddux, MD, MSCS; Peter M. Mourani, MD.

Connecticut: Connecticut Children's, Hartford. Christopher L. Carroll, MD, MS.

Connecticut: Yale New-Haven Children's Hospital, New Haven. John S. Giuliano, Jr., MD.

Florida: Holtz Children's Hospital, Miami. Gwenn E. McLaughlin, MD, MSPH.

Georgia: Children's Healthcare of Atlanta at Egleston, Atlanta. Keiko M. Tarquinio, MD.

Illinois: Ann & Robert H. Lurie Children's Hospital of Chicago, Chicago. Kelly N. Michelson, MD, MPH; Bria M. Coates, MD.

Indiana: Riley Hospital for Children, Indianapolis. Courtney M. Rowan, MD, MS.

Iowa: University of Iowa Stead Family Children’s Hospital, Iowa City. Kari Wellnitz, MD; Guru Bhoojhawon MBBS, MD.

Kentucky: University of Louisville and Norton Children’s Hospital, Louisville, Janice E. Sullivan, MD; Vicki L.Montgomery, MD; Kevin M. Havlin, MD.

Louisiana: Children's Hospital of New Orleans, New Orleans. Tamara T. Bradford, MD.

Maryland: Johns Hopkins Children’s Hospital, Baltimore. Becky J. Riggs,MD; Melania M. Bembea, MD, MPH, PhD.

Maryland: University of Maryland Children’s Hospital, Baltimore. Ana Lia Graciano, MD.

Maryland: Sinai Hospital of Baltimore, Baltimore. Susan V. Lipton, MD, MPH.

Massachusetts: Baystate Children’s Hospital, Springfield. Kimberly L. Marohn, MD.

Massachusetts: Boston Children’s Hospital, Boston. Adrienne G. Randolph, MD; Margaret M. Newhams, MPH; Sabrina R. Chen; Cameron C. Young; Suden Kucukak, MD; Katherine Kester; Jane W. Newburger, MD, MPH; Kevin G. Friedman, MD; Mary Beth F. Son, MD; Janet Chou, MD.

Massachusetts: Mass General Hospital for Children, Boston. Ryan W. Carroll, MD, MPH; Phoebe H. Yager, MD; Neil D. Fernandes, MBBS.

Michigan: Children’s Hospital of Michigan, Detroit. Sabrina M. Heidemann, MD.

Michigan: University of Michigan CS Mott Children’s Hospital, Ann Arbor. Heidi R. Flori, MD, FAAP.

Minnesota: University of Minnesota Masonic Children’s Hospital, Minneapolis, Janet R. Hume, MD, PhD.

Minnesota: Mayo Clinic, Rochester. Emily R. Levy, MD.

Mississippi: Children’s Hospital of Mississippi, Jackson. Charlotte V. Hobbs, MD.

Missouri: Children’s Mercy Hospital, Kansas City. Jennifer E. Schuster, MD.

Missouri: Washington University in St. Louis. Philip C. Spinella MD.

Nebraska: Children’s Hospital & Medical Center, Omaha. Melissa L. Cullimore, MD, PhD; Russell J. McCulloh, MD.

New Jersey: Hackensack University Medical Center, Hackensack. Katharine N. Clouser, MD.

New Jersey: Newark Beth Israel Medical Center, Newark. Rowan F. Walsh, MD

New Jersey: Bristol-Myers Squibb Children's Hospital, New Brunswick. Lawrence C. Kleinman, MD, MPH,

FAAP; Simon Li, MD, MPH; Steven M. Horwitz, MD.

New Jersey: St. Barnabas Medical Center, Livingston. Shira J. Gertz, MD.

New York: Golisano Children’s Hospital, Rochester. Kate G. Ackerman, MD; Jill M. Cholette, MD.

New York: Kings County Hospital, Brooklyn. Michael A. Keenaghan, MD.

New York: Maria Fareri Children's Hospital, Valhalla. Aalok R. Singh, MD.

New York: The Mount Sinai Hospital, New York City. Sheemon P. Zackai, MD; Jennifer K. Gillen, MD.

New York: Hassenfeld Children’s Hospital at NYU Langone, New York. Adam J. Ratner, MD, MPH; Heda

Dapul, MD; Vijaya L. Soma, MD.

New York: Stony Brook University Hospital, Stony Brook. Ilana Harwayne-Gidansky, MD; Saul R. Hymes, MD.

New York: SUNY Downstate Medical Center University Hospital, Brooklyn. Sule Doymaz, MD.

North Carolina: University of North Carolina at Chapel Hill, Chapel Hill. Stephanie P. Schwartz, MD; Tracie C.

Walker, MD.

Ohio: University Hospitals Rainbow Babies and Children's Hospital, Cleveland. Steven L. Shein, MD; Amanda

N. Lansell, MD.

Ohio: Nationwide Children’s Hospital, Columbus. Mark W. Hall MD, FCCM.

Ohio: Cincinnati Children’s Hospital, Cincinnati. Mary A. Staat, MD, MPH.

Pennsylvania: Children’s Hospital of Philadelphia, Philadelphia. Julie C. Fitzgerald, MD, PhD, MSCE; Jenny L.

Bush RN, BSN; Ryan H. Burnett, BS.

Pennsylvania: Penn State Children’s Hospital, Hershey. Neal J. Thomas, MD, MSc.

Pennsylvania: St. Christopher’s Hospital for Children, Philadelphia. Monica L. Koncicki, MD.

Pennsylvania: UPMC Children’s Hospital of Pittsburgh. Ericka L. Fink, MD, MS; Joseph A. Carcillo, MD.

South Carolina: MUSC Children’s Health, Charleston. Elizabeth H. Mack, MD, MS.; Laura Smallcomb, MD.

Tennessee: Monroe Carell Jr. Children’s Hospital at Vanderbilt, Nashville. Natasha B. Halasa, MD, MPH.

Tennessee: Le Bonheur Children’s Hospital, Memphis. Dai Kimura, MD.

Texas: Texas Children's Hospital, Houston. Laura L. Loftis, MD.

Texas: University of Texas Health Science Center, Houston. Alvaro Coronado Munoz, MD.

Texas: University of Texas Southwestern, Children's Medical Center Dallas, Dallas. Mia Maamari, MD; Cindy Bowens, MD, MSCS.

Utah: Primary Children's Hospital, Salt Lake City. Hillary Crandall, MD, PhD.

Washington: Seattle Children's Hospital, Seattle. Lincoln S. Smith, MD; John K. McGuire, MD.

CDC COVID-19 Response Team on Overcoming COVID-19: Manish M. Patel, MD, MPH; Leora R. Feldstein, PhD, MSc; Mark W. Tenforde, MD PhD; Ashley M. Jackson MPH; Nancy Murray MSc; Charles E. Rose, PhD.

References

1. Stoeckius M, Zheng S, Houck-Loomis B, Hao S, Yeung BZ, Mauck WM, 3rd, et al. Cell Hashing with barcoded antibodies enables multiplexing and doublet detection for single cell genomics. *Genome Biol.* 2018;19(1):224.
2. Zheng GX, Terry JM, Belgrader P, Ryvkin P, Bent ZW, Wilson R, et al. Massively parallel digital transcriptional profiling of single cells. *Nat Commun.* 2017;8:14049.
3. Hao Y, Hao S, Andersen-Nissen E, Mauck WM, 3rd, Zheng S, Butler A, et al. Integrated analysis of multimodal single-cell data. *Cell.* 2021;184(13):3573-87 e29.
4. Korsunsky I, Millard N, Fan J, Slowikowski K, Zhang F, Wei K, et al. Fast, sensitive and accurate integration of single-cell data with Harmony. *Nat Methods.* 2019;16(12):1289-96.
5. Liberzon A, Birger C, Thorvaldsdottir H, Ghandi M, Mesirov JP, and Tamayo P. The Molecular Signatures Database (MSigDB) hallmark gene set collection. *Cell Syst.* 2015;1(6):417-25.
6. Schmitz-Abe K, Li Q, Rosen SM, Nori N, Madden JA, Genetti CA, et al. Unique bioinformatic approach and comprehensive reanalysis improve diagnostic yield of clinical exomes. *Eur J Hum Genet.* 2019;27(9):1398-405.
